# The immunomodulatory impact of naturally derived neem leaf glycoprotein on the initiation progression model of 4NQO induced murine oral carcinogenesis: a preclinical study

**DOI:** 10.3389/fimmu.2024.1325161

**Published:** 2024-03-22

**Authors:** Juhina Das, Saurav Bera, Nilanjan Ganguly, Ipsita Guha, Tithi Ghosh Halder, Avishek Bhuniya, Partha Nandi, Mohona Chakravarti, Sukanya Dhar, Anirban Sarkar, Tapasi Das, Saptak Banerjee, Sandip Ghose, Anamika Bose, Rathindranath Baral

**Affiliations:** ^1^ Department of Immunoregulation and Immunodiagnostics, Chittaranjan National Cancer Institute, Kolkata, India; ^2^ Department of Oral Pathology, Dr. R. Ahmed Dental College and Hospital, Kolkata, India; ^3^ Department of Pharmaceutical Technology (Biotechnology), National Institute of Pharmaceutical Education and Research (NIPER), Sahibzada Ajit Singh Nagar, Punjab, India

**Keywords:** 4NQO, CD8^+^ T cells, immunotherapeutics, neem leaf glycoprotein, Notch1, oral carcinogenesis, Stat3, epithelial mesenchymal transition

## Abstract

**Introduction:**

Murine tumor growth restriction by neem leaf glycoprotein (NLGP) was established in various transplanted models of murine sarcoma, melanoma and carcinoma. However, the role of NLGP in the sequential carcinogenic steps has not been explored. Thus, tongue carcinogenesis in Swiss mice was induced by 4-nitroquinoline-1-oxide (4NQO), which has close resemblance to human carcinogenesis process. Interventional role of NLGP in initiation-promotion protocol established during 4NQO mediated tongue carcinogenesis in relation to systemic immune alteration and epithelial-mesenchymal transition (EMT) is investigated.

**Methods:**

4NQO was painted on tongue of Swiss mice every third day at a dose of 25µl of 5mg/ml stock solution. After three consecutive treatments with 4NQO (starting Day7), one group of mice was treated with NLGP (s.c. 25μg/mice/week), keeping a group as PBS control. Mice were sacrificed in different time-intervals to harvest tongues and studied using histology, immunohistochemistry, flow-cytometry and RT-PCR on different immune cells and EMT markers (e-cadherin, vimentin) to elucidate their phenotypic and secretory status.

**Results:**

Local administration of 4NQO for consecutive 300 days promotes significant alteration in tongue mucosa including erosion in papillae and migration of malignant epithelial cells to the underlying connective tissue stroma with the formation of cell nests (exophytic-hyperkeratosis with mild dysplasia). Therapeutic NLGP treatment delayed pre-neoplastic changes promoting normalization of mucosa by maintaining normal structure. Flow-cytometric evidences suggest that NLGP treatment upregulated CD8^+^, IFNγ^+^, granzyme B^+^, CD11c^+^ cells in comparison to 4NQO treated mice with a decrease in Ki67^+^ and CD4^+^FoxP3^+^ cells in NLGP treated cohort. RT-PCR demonstrated a marked reduction of MMP9, IL-6, IL-2, CD31 and an upregulation in CCR5 in tongues from 4NQO+NLGP treated mice in comparison to 4NQO treated group. Moreover, 4NQO mediated changes were associated with reduction of e-cadherin and simultaneous up-regulation of vimentin expression in epithelium that was partially reversed by NLGP.

**Discussion:**

Efficacy of NLGP was tested first time in sequential carcinogenesis model and proved effective in delaying the initial progression. NLGP normalizes type 1 immunity including activation of the CD8^+^T effector functions, reduction of regulatory T cell functions, along with changes in EMT to make the host systemically alert to combat the carcinogenic threat.

## Graphical Abstract

Topical application of 4NQO on mouse tongues enhances carcinogenic events, like, angiogenesis, proliferation and epithelial-mesenchymal transition, along with invasive properties. Therapeutic application of Neem Leaf Glycoprotein (NLGP) hinders preneoplastic events and delays carcinogenesis. Immunological study deciphers the role of augmented CD8^+^T cells and lowered Treg population finally acting on Notch1-Stat3 pathway leading to the restriction in carcinogenesis.

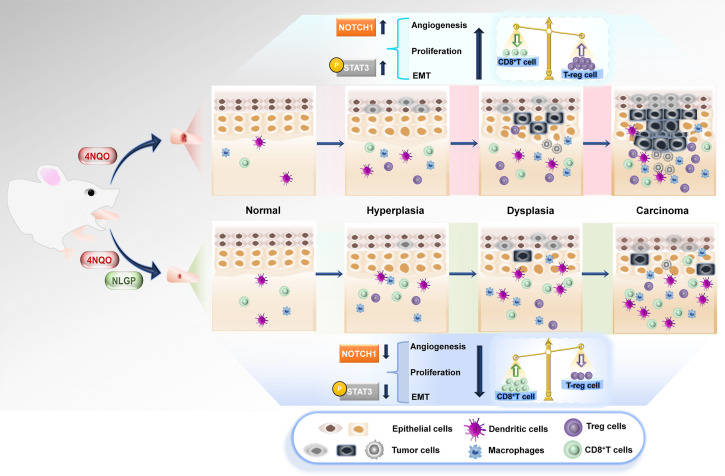

## 1 Introduction

Restriction of growth of different murine solid tumors, like, melanoma (1), carcinoma (2), sarcoma (3) and lymphoma (*unpublished observation*) by neem (*Azadichrata indica*) leaf glycoprotein (NLGP) is widely discussed. These transplantable tumor xenografts grow subcutaneously in linear fashion and therapeutic efficacy of natural nontoxic immunomodulator, NLGP, have been tested in these tumor models (4). Observed tumor inhibition by NLGP is chiefly due to correction of altered dendritic cell-T cell functionality (5–8), suppression of suppressor cells (9, 10) and optimization of Th1-Th2 cytokine-chemokine loop (11, 12). However, human oral squamous cell carcinogenesis (OSCC), follow the initiation-promotion protocol having multiple stages, like, hyperplasia, dysplasia, carcinoma *in situ* etc (13). Unfortunately, this multistage carcinogenesis protocol could not be visualized in tested murine solid tumor models. Thus, the necessity is always felt to check the tumor growth restricting ability of NLGP in multistep carcinogenesis model having close resemblance to initiation-progression of human cancer.

The most widely used model of induced experimental oral (tongue) carcinogenesis that may fulfill our requirement is the usage of 4-nitroquinoline-1-oxide or 4NQO (14, 15). 4NQO is a synthetic water soluble carcinogen, causes several systemic and molecular damages by promoting DNA adduct formation and intracellular oxidative stress (16). A close association between chewing betel nuts and human oral cancers is well established, particularly in OSCC prone eastern India (17). This 4NQO induced model mimics the sequential steps of oral carcinogenesis (18), namely, hyperplasia, dysplasia and carcinoma *in situ*, which occur in humans as a result of chronic tobacco abuse (19).

In the present communication, we have tested anti-carcinogenic effect of NLGP on 4NQO induced tongue carcinogenesis model and examined the role of NLGP in maintaining optimum homeostasis between effector and suppressor immune functions. Obtained results suggest the critical role of NLGP in slowing down the preneoplastic changes in tongue mucosa that eventually inhibits the tumor formation on mouse tongue principally by activating CD8^+^T cells and suppressing regulatory T cells.

## 2 Materials and methods

### 2.1 Mice

Female Swiss mice MGI:5652853 (n=90, in each group) (Age: 4–6 weeks; Body weight: 24–27 g) were obtained from the Institutional Animal Care and Maintenance Department, Chittaranjan National Cancer Institute, Kolkata with prior approval from Institutional Animal Ethics Committee (IAEC Ref No. 1774/RB-11/2016/7). Initially mice of both sexes were used for the experiment. Prolonged 4NQO exposure causes aggression among male mice which reduces their survivability. With suggestion from IAEC, final experiments were conducted on female mice only. Moreover, to avoid the interference of hormonal difference only female mice were included in the study. Autoclaved dry pellet diet (Epic Laboratory Animal Feed, Kalyani, India) and water were given *ad libitum.*

### 2.2 Neem leaf glycoprotein

An extract from neem (*Azadirachta indica*) leaves was prepared by the method as described previously (20, 21). Matured deep green neem leaves of same size and color (indicative of same age), were collected in summer (April-May) from Salt Lake area of Kolkata, India. These leaves were shed-dried and pulverized. Leaf powder was soaked overnight in PBS, pH 7.4. The supernatant was collected by centrifugation at 1500 rpm, extensively dialyzed against PBS, pH 7.4 and concentrated by Centricon membrane filter (Millipore Corporation, MA, USA) with a 10 KDa MW cut-off. NLGP purity was checked by Size Exclusion-HPLC (SE-HPLC) in a protein PAK 300 SW column (8). The protein concentration was measured by Lowry’s method (22). NLGP was injected subcutaneously (s.c.) in the right flank of mice at a concentration of 25µg/mice/week for a period of 4 weeks, then once in every 30 days.

### 2.3 Oral carcinogenesis

For stock solution, 4NQO powder (Sigma N8141-5G) was dissolved in autoclaved distilled water at a concentration of 5mg/ml (23, 24) and stored at -20°C till use in aliquots. Mice tongues were painted with 4NQO (25μl) every third day using sterile cotton buds. A group of mice in each set was kept as control, painted similarly with PBS, pH 7.4. Animals were refrained from drinking water for 3 hrs post 4NQO application to ensure total absorption of the carcinogen. Animal death and abnormal symptoms, if any, were recorded. Animals were separated in special cages with fresh bed when features of suffering were appeared and euthanized, whenever necessary, under supervision of Institutional veterinarian.

### 2.4 Mice body weight, general health and physical behavior

The health of animals was monitored twice a day in every working day and once in holidays. Swiss albino female mice, with no visible abnormalities or injuries, were selected. All mice displayed healthy appetite and drinking patterns along with active movement in experiment initiation. Mice body weight, general health and physical behavior were noted in all 4NQO and 4NQO+NLGP mice cohorts. The mice tongues were observed for any visible abnormalities and presence of flaky skin. Other structural abnormalities, like, body hair loss, facial swelling along with reddish patches in and around the auricular areas, were noted regularly.

### 2.5 Surgical removal of mice tongues

Mice were euthanized by overdose of ketamine HCL (160 mg/kg) + xylazine (20 mg/kg) intraperitoneally (i.p) as per CPCSEA (Committee for the Purpose of Control and Supervision of Experiments on Animals) guidelines on various days of early (up to 100 days), intermediate (101-200 days) and late (201-300 days) phases or when animal looks sick or any tumor necrosis was occurred. Immediately after sacrifice, the tongues were harvested with sharp scalpel, examined for the presence of gross lesions and photographed. All animal experiments were carried out as per ARRIVE guidelines (25).

### 2.6 Tongue histopathology

Harvested tongues were washed thoroughly with PBS, fixed in 10% formal-saline for 24-48 hrs, embedded in paraffin and sectioned (5-6μm) with Rotary microtome (Leica, Germany). Sections were stained with hematoxylin and eosin (H&E) as per standard protocol (26). Mounted sections were studied under bright field microscope (Axiovision 105 Carl-Zeiss, Germany), image was captured under Zen 2.0 software, and every change was noted and photographed. The histopathological assessment is scored based on the criteria described in the article, PMID: 18251935 (27).

### 2.7 Immunohistochemistry

Sections were stained for e-cadherin (BD Biosciences Cat# 610181) and vimentin (BD Biosciences Cat# 550513), at a concentration of 1:200 as per method described (28), using HRP tagged secondary antibodies. Briefly, sections were deparaffinized in xylene for 10 mins followed by rehydration in alcohol gradients. Endogenous peroxidase was blocked by treatment with 3% H_2_O_2_ (Merck 17544) in methanol for 30 mins, following rehydration. Antigen retrieval was performed in 1 mM sodium citrate buffer at 100°C for 15 mins followed by cooling at RT for 15 mins. Blocking of non-specific sites was carried out by incubating the slides for 30 mins in 8% BSA-PBS. Sections were incubated overnight with primary antibodies diluted in 1% BSA followed by washing with PBS-Tween 20 and incubation with HRP tagged anti-mouse secondary antibodies. Chromogenic color was developed using AEC Substrate (Vector Laboratory SK4200) as per manufacturer’s protocol. The sections were counterstained with Hematoxylin (Merck-HX68597049) for 40 secs and mounted with Vectamount (Vector Laboratories H5501). Image acquisition was done on Carl Zeiss Plan Achromat bright field microscope, Axiocam 1058 color camera with objectives 4x, 10x and 20x.

### 2.8 Preparation of single cells

The isolated tongues were thoroughly washed with PBS to clear off any blood and tissue remains. They were then chopped carefully using sterile scissors and forceps. The chopped tongues were allowed to incubate in collagenase (1 mg/ml) for a span of 4-6 hrs for tissue digestion, then were smashed using sterile PBS and passed through a 75 mm strainer. The solution was then aliquoted into vials and stored with 95% freezing mixture for future work.

### 2.9 Flow-cytometric analysis

For flow-cytometric analysis, cells (2×10^5^) were stained with different FITC (NB-720-F-IMG, 1:200) tagged CD8 (#clone 53-6·7, E-biosciences#140081-82,1:200), CD4 (clone#GK1.5, E-biosciences, #140081-45,1:200), CD25 (1:200, BioLegend, Cat#102005), Ly6G (1:200, BioLegend, Cat#108406), CD11b (1:200, BioLegend, Cat# 101208), CD11C (1:200, BioLegend, Cat#117305), IFNγ (clone R4-6A2, #14-7312-85, E-biosciences, 1:200) and Ki-67 (clone#16A8, 1:200, #652401; Biolegend) and PE-tagged CD11b (1:200, BD Biosciences, Cat#101207), Foxp3 (1:200, BioLegend, Cat# 320002), Granzyme B (#2505, Santa Cruz Biotechnology, Cat#Sc8022,1:500) and NK1.1 (BD Biosciences, Cat#553164), CD31 (BioLegend, Cat#102401), VEGF (Santa Cruz Biotechnology, Cat# sc-507), Notch 1 (Santa Cruz Biotechnology, Cat# sc-373891), DLL-1 (1:500, Santa Cruz Biotechnology, Cat# sc-8155), Stat3 (1:500, BioLegend, Cat#624601) and Jagged 1 (1:500, Santa Cruz Biotechnology, Cat#sc-6011) antibodies. After labeling, the cells were washed with FACS buffer (PBS with 1% FBS). Similarly, intracellular molecules, Granzyme B (1:1,000, BD Biosciences, Cat#558132, Perforin (E-biosciences, Cat#11-9392-82) and Foxp3 (BioLegend, Cat#102005) were stained with mentioned antibodies after permealization with Perm-buffer (0·1% Saponin in BSA). The cells were fixed after staining with 1% paraformaldehyde in PBS and cytometry was performed with Cell-Quest software on a FACS Caliber (Becton Dickinson, Mountainview, CA). Suitable negative isotype controls were used to rule out the background fluorescence. The percentage of each positive population and MFI were determined using quadrant statistics. Data was analyzed by either Cell-Quest and or FlowJo software (Beckton Dickinson, Mountain view, CA). For flow-cytometric analysis of Ki67^+^ proliferating cells, the cells were washed with PBS and then permeabilized using 70% methanol to stain with FITC tagged anti-Ki67 (1:500, BioLegend, Cat#652401) antibody. Detailed list of reagents and antibodies used have been provided in **Supplementary Table 1**.

### 2.10 RNA isolation and RT-PCR

Cellular RNA was isolated using Trizol (Invitrogen, CA, USA) and random hexamers were used to generate corresponding cDNA (First Strand cDNA Synthesis Kit; Fermentas, MD, USA). The RNA concentration was evaluated by absorbance readings at 260 nm using a NanoDrop spectrophotometer (Thermo Scientific, Fremont, CA, USA). Amplification was performed using 2X Go Taq Green Mix (Promega, WI, USA) and PCR was carried out with gene-specific primer listed in **Supplementary Table 2** with the following program: 94°C for 5 min; 35 cycles of 94°C for 30 s, 54-57°C for 30 s and 72°C for 1 min and 72°C for 5 min. PCR products were identified by image analysis software for gel documentation on ChemiDoc XRS+ (BioRad Laboratories, CA, USA) after electrophoresis on 1.5% agarose gels and staining with ethidium bromide. Following acquisition, band intensities were measured on Image Lab software V5·1 for the mean pixel intensities. The relative intensity of each gene was calculated by dividing the intensity of a given gene to the intensity value of its corresponding GAPDH gene. Detail of all primers along with their accession numbers and Tm has been provided in **Supplementary Table 2.**

### 2.11 Immunofluorescence and confocal-microscopy

Immunofluorescence staining was performed on paraffin embedded tongue tissue sections to detect different cells expressing e-cadherin (1:50, BD Biosciences, Cat# 610181), vimentin (1:150, BD Biosciences, Cat# 550513), CD8 (1:200, BD Biosciences, Cat#553033), GrB (1:150, E-biosciences, Cat#12889880), IFNγ (1:150, Ebioscience, Cat #505808), Notch 1 (Santa Cruz Biotechnology, Cat#SC373891), MMP9 (1:50, Santa Cruz Biotechnology, Cat#SC6840), Stat3 (1:100, BioLegend, Cat# 678002) and pStat3 (1:100, BioLegend, Cat#651002). Briefly, sections were deparaffinized in xylene for 15 mins followed by rehydration in descending concentrations of alcohol for 10 mins each. Antigen retrieval was performed in 10mM sodium citrate buffer at 94°C for 20 mins. For intracellular staining, permeabilization was performed by 0.1% Triton X in PBS for 10 mins. Blocking was performed in 6% BSA for 2-3 hrs followed by primary antibody and left overnight at 4°C. In case of purified primary antibody, PE (1:500, BD Biosciences, Cat#550589) and FITC (1:500, Novus Biologicals, NB-720-F-IMG) conjugated secondary antibodies were used and incubated for 2-3 hrs. The sections were washed with PBS-Tween 20 to remove all traces of unbound antibodies. The cells were finally mounted with Fluoroshield™ DAPI for nuclear staining and observed under fluorescence microscope (BX53, Camera-XM10, Olympus Life Sciences, Tokyo, Japan). Image analysis was done on FiJi, Image J (RRID: SCR_003070) software. Details of all reagents including antibodies are provided in **Supplementary Table 1**.

For confocal-microscopy, representative slides from day 300 tongues of normal, 4NQO and 4NQO+NLGP treated mice were stained for VEGF (1:200, BD Biosciences, Cat#553033). The slides were mounted with DAPI and observed for probable angiogenesis under confocal microscope (Leica microsystems, Stellaris 5.0).

### 2.12 Protein-protein interaction search

To understand the interactions between all the different proteins involved in this study, we used database String (Search Tool for the Retrieval of Interacting Genes/Proteins), Version8.0 RRID: SCR_005223 (29), which relies on the annotated proteomes maintained by Swissprot. These interactions apart from being physical in nature can also be of functional importance. Each protein in this study has been assigned a color, and the interaction between the other proteins involved in this study is represented by multicolored lines. Each of these colors represents a type of interaction, mentioned in the legend. Each of these interactions have different coefficient of interaction listed separately in **Supplementary Table 3**. Each of our protein levels used in this experiment has undergone a modulation after 4NQO and 4NQO+NLGP treatment. The corresponding changes with the treatment have been identified as arrows (Green for up regulated expression and Red for down regulated expression).

### 2.13 Database searches

To correlate our mouse-based results, we needed to explore the same findings in human subjects. A random database search was done (30) to find out the probable databases that could be of interest to this study. Data or most mutated genes of human OSCC was obtained from TCGA (RRID: SCR_003193) database (31). Human immunological data showing relevance of CD8^+^T cells, macrophages and dendritic cells in relation to OSCC (normal, HPV^+^, HPV^-^) patients were obtained from TIMER 2.0 RRID : SCR_018737 (Tumor Immuno Estimation Resource) (32). The data of percentage of human genes mutated along with Notch1 in OSCC patients and Stat3 mutations status were analyzed using GEPIA RRID : SCR_018294 (Gene Expression Profiling Interactive Analysis 2.0) (33) database. Box plots indicating correlation of NOTCH1 in relation to CD8a and Twist 1 was also obtained by correlation expression Analysis on Gepia2.0 database. tSNE plots about Notch 1 and Stat3 expression on murine tongues were obtained from Tabula muris database (34).

### 2.14 Statistical analysis

All results represent the average of 3 independent *in vivo* experiments. The number of experiments is stated in the results section and legends to Figurers. To ensure that the data follows a normal distribution pattern, normality and log normality tests were performed. All of the data passed the Shapiro-Wilk test of normal distribution. For all assays, a value represents the mean of 6 individual observations in each time point per group and is presented as mean ± SD. All pairs of columns were compared using two-way ANOVA followed by Tukey’s multiple comparison test with Graphpad Prism (Version 8.4.2) software (GraphPad software, San Diego, CA, USA).

## 3 Results

### 3.1 NLGP maintains macroscopical tongue features during 4NQO mediated carcinogenesis

**Figure 1:**
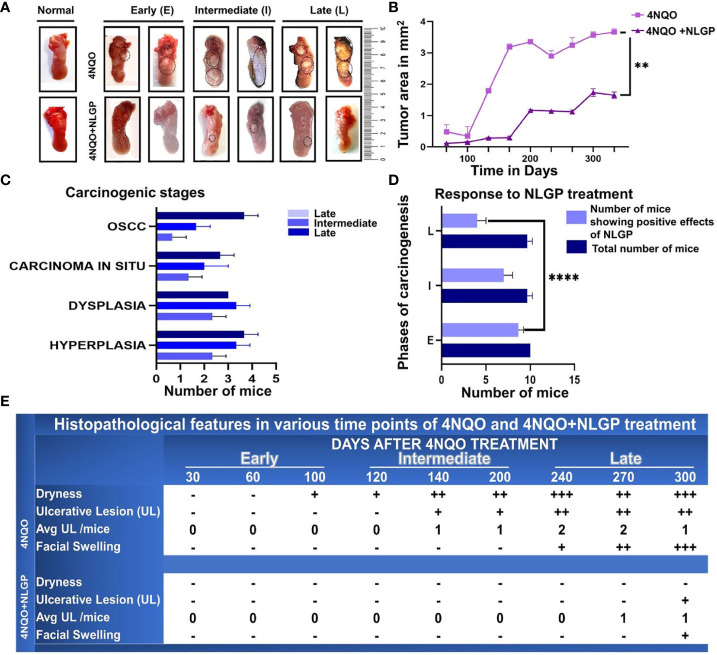
NLGP maintains macroscopical tongue features during 4NQO mediated carcinogenesis. **(A)** Representative images of mouse tongues at early (day 1-day 100), intermediate (day101-day 200) and late stages (day 201- day 330) of 4NQO and 4NQO+NLGP treated tongues. Dotted areas indicate the presence of lesions. **(B)** Line graphs representing the mean lesion area in mm2. Data presented as mean ± SD. **(C)** Bar diagram representing total number of mice collectively from the experimental (4NQO) group, displaying different stages of carcinogenesis in each of early, intermediate and late phases (n=30), inclusive of all phases, experiment repeated thrice. **(D)** Total number of mice that was benefited by NLGP treatment in early, intermediate and late phases n=3 at each time point. **(E)** Table indicating changes appearing in mouse tongues and faces at different phases of carcinogenesis. **p< 0.0001,+ minimally present, ++ present in moderate levels, +++ present in high levels, -, absent.

A series of macroscopic alterations precede the carcinogenic events, including appearance of white and red patches (leukoplakia and erythroplakia) respectively (35), which were observed in this experiment around 120 days of the first 4NQO exposure (**Figure 1A**). Intensification of such features eventuated around day 200 with a blackened appearance on the exterior. At the experimental end point, most animals exhibited tumor formation in the 4NQO group (15/30 in the intermediate phase and 25/30 in late phase) and that maximizes at around day 300, the rest displaying initial carcinogenic stages. However, mice having NLGP therapy have shown almost normal tongue (**Figure 1B**) with mild black appearance in later phase. Of 30 mice in the early stage, we mostly observed hyperplasia and dysplasia. Cases of OSCC in late stage of 4NQO cohort have shown 13 times increase as compared to intermediate stages (**Table 1**). Hyperplastic and dysplastic changes were observed to be maximal in the early and intermediate phases than late phase of carcinogenesis (**Figure 1C**). Of the 90 mice receiving NLGP immunotherapy, 69 mice were reported healthy across the three time points (**Figure 1D**; **Table 2**), of which 49 belonged to the early and intermediate phases. **Figure 1E** represents comparative changes across both 4NQO and 4NQO+NLGP cohorts.

**Table 1:** Number of mice affected by 4NQO treatment in experimental (4NQO) and **t**herapeutic (4NQO+NLGP) groups.

	Early		Intermediate		Late	
**Carcinogenic features**	**4NQO** 30 (n=10 per repeat)	**4NQO + NLGP** 30 (n=10 per repeat)	**4NQO** 30 (n=10 per repeat)	**4NQO + NLGP** 30 (n=10 per repeat)	**4NQO** 30 (n=10 per repeat)	**4NQO + NLGP** 30 (n=10 per repeat)
**Hyperplasia**	9	1	5	1	2	2
**Dysplasia**	11	3	6	3	9	1
**Carcinoma *in situ***	5	0	8	3	5	4
**OSCC**	1	0	5	0	10	2
**Total affected mice at the end of all experimental repeat in each phase**	**26**	**3**	**24**	**7**	**26**	**9**

**Table 2:** Number of responding mice to NLGP therapy.

Phases of Carcinogenesis	Total number of mice			Mice responded to NLGP therapy		
Early	10	10	10	10	8	9
Intermediate	10	10	10	8	8	6
Late	10	9	10	8	7	5
**Total**	**30**	**29**	**30**	**26**	**23**	**20**

### 3.2 NLGP protects detoriation of physical features and body weight loss during 4NQO mediated tongue carcinogenesis

**Figure 2:**
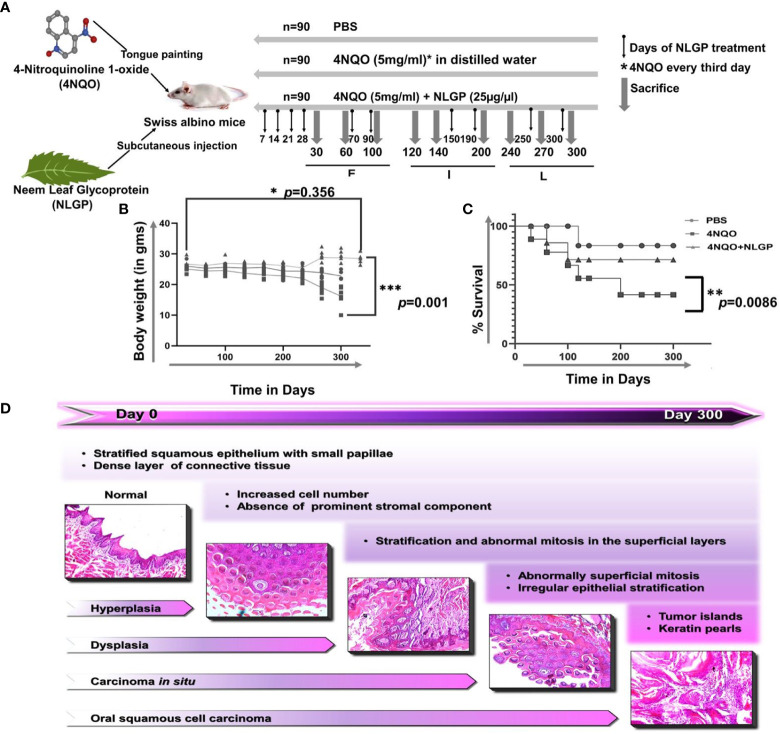
NLGP protects detoriation of physical features and body weight loss during 4NQO mediated tongue carcinogenesis. **(A)** 4NQO, NLGP and treatment protocol. Experimental design is showing 4NQO and NLGP treatment. 4NQO was applied by tongue painting every third day and NLGP was given subcutaneously (25μg/mice) on day 7, 14, 21 and 28. Thereafter, NLGP dose was repeated on days 100, 150, 200, 250 and 300. **(B)** Graphical representation of body weight changes after 4NQO painting with and without NLGP treatment, n=9 per group. Analysis was done by 2 Way ANOVA followed by Tukey’s multiple test. **(C)** Kaplan Meyer’s survival analysis for control, 4NQO and 4NQO+NLGP treated mice. Significance was obtained by Log-rank (Mantel-Cox) test. **p=0.0086. **(D)** Representation of sequential carcinogenesis stages from day 0 to day 300. H&E images were captured on 20x magnification. *p<0.001.

One of the most life-threatening and incapacitating effects of cancer is cachexia. Cachexia results from a complicated interplay between the tumor and the host and is accompanied by anorexia, waste of fat and muscle tissue, psychological discomfort, and a decreased quality of life (36). Similar to cancer patients the same observations were recapitulated in the experimental set up. 4NQO was applied to the tongues of Swiss albino mice over a span of 300 days and examined once every 30 days for progression of carcinogenesis. Therapeutic application of NLGP was started after the 7^th^ day of 4NQO exposure and continued every 8^th^ day for the first 30 days (**Figure 2A**). Examination of their physical features revealed, with increasing number of days of 4NQO treatment mice appeared weaker, lethargic with low appetite and showed slower movement. At around day 100, a reduction in the appetite was seen as assessed by the amount of uneaten pellets every day. Even though mice of the control and 4NQO+NLGP groups had normal dietary intake, there was a marked reduction of food intake in the 4NQO cohort. A mice group showed patchy skin appearance due to hair loss and this feature appeared generally150 days onwards. Modest swelling and reddening of the facial region was observed. On contrary, mice from NLGP cohort acquired reversal of observed features after 2 weeks of NLGP administration (**Supplementary Figure 1D**). NLGP treated mice were comparatively active, mobile having normal food intake. Body weight measurement exhibited enormous loss in mice receiving 4NQO alone with 50% reduction (23gm to 12gm) in some terminal cases. On the other hand, in NLGP cohort no weight loss was recorded, in spite of that weight gain in few cases was noted (**Figure 2B**), including an example of weight gain upto 29 gms (**Figure 2B**). As shown in **Figure 2C**, some mice of 4NQO group died after a few initial 4NQO doses; however, no mortality was recorded within stipulated time frame in NLGP treated 4NQO group. At experimental end point (day 300), most of the mice from NLGP cohort was active and healthy. However, at the same time point 50% mice died in 4NQO cohort (**Figure 2C**). The stage wise histological representation from a normal to 4NQO affected tongue with advancement of carcinogenesis have been represented in **Figure 2D**.

### 3.3 NLGP prevents progression of pre-neoplastic histopathology during 4NQO mediated tongue carcinogenesis

**Figure 3:**
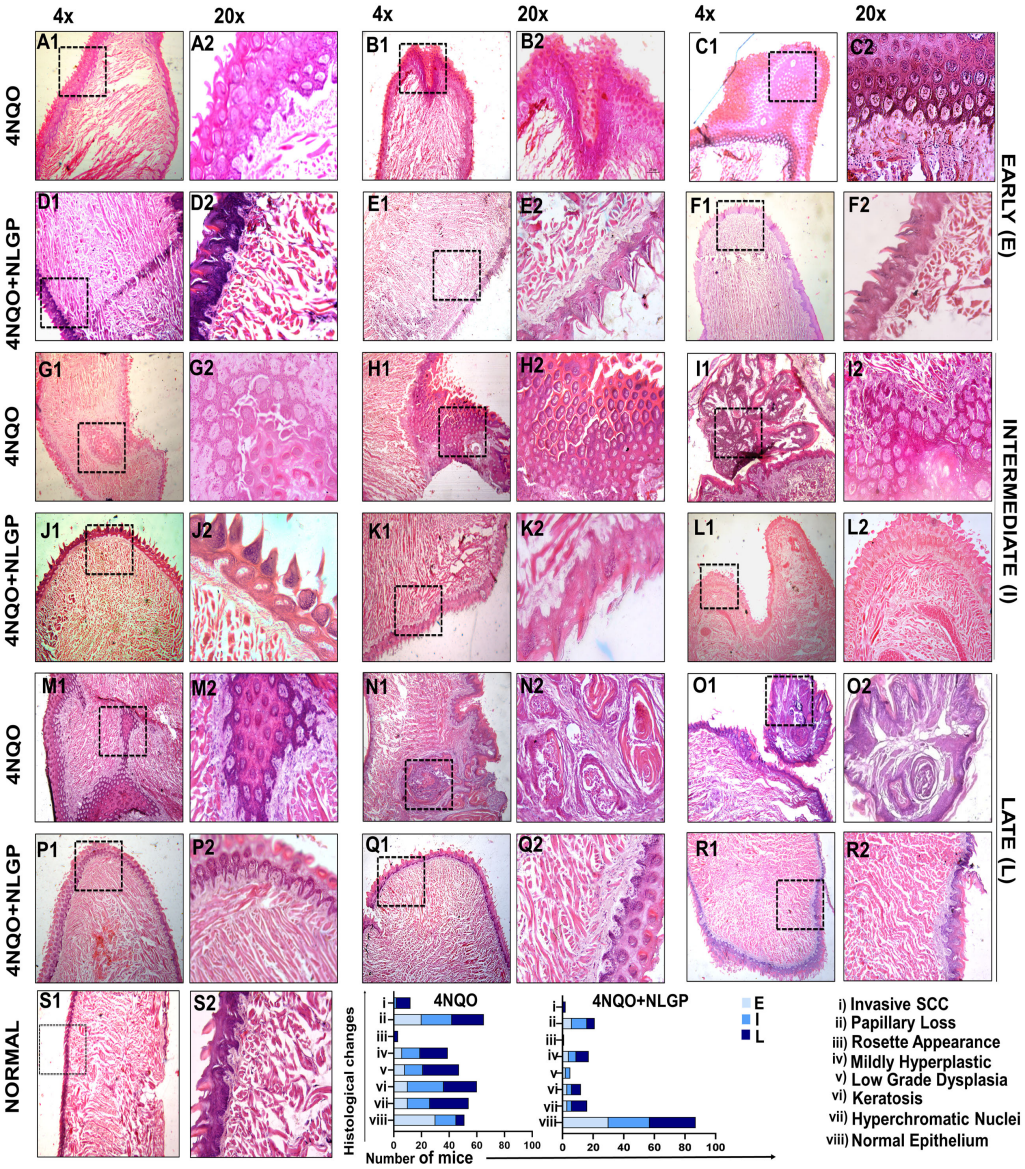
Histopathological features of 4NQO painted mice tongues in different stages of carcinogenesis with or without NLGP treatment. Swiss mice tongues were painted with 4NQO with or without NLGP in early **(A–F)**, intermediate **(G–L)** and late **(M–R)** phases, along with normal tongue features as labeled **(S)**. 4NQO painted tongues are shown in **A–C**, **G–I**, **M–O** and those treated with NLGP are presented in **D**–**F**, **J–L**, **P–R**. Columns 1,3,5 are in 4× (50µm) and columns 2,4,6 are in 20× (20µm) magnification. Each row shows 3 representations from each group. Bars in lower panel present histological alterations in 4NQO and 4NQO+NLGP cohorts in early, intermediate and late phases of the experiments (n=3 per group).

Histopathological analysis revealed that 4NQO treated mice exhibited sequential stages of initiation-promotion protocol of oral carcinogenesis. Changes in the epithelium as observed in different days of carcinogenesis were divided into several categories, like, normal epithelium, hyperplasia, dysplasia (mild, moderate, severe) and carcinoma *in situ*. On day 30, 4NQO treated mice showed mild hyperplastic epithelium. On the other hand, mice of 4NQO+NLGP cohort exhibited normal mucosal pattern. Papillary structure of the tongue epithelium was maintained in both groups. On day 60 onwards, papillary structure of the tongue showed degenerative changes in 4NQO treated mice, whereas, in 4NQO+NLGP cohort no such papillary loss was observed. Thickening of the epithelium with mild to moderate dysplasia was noted (**Figures 3A–C**) in 4NQO group with no significant dysplastic changes in NLGP cohort (**Figures 3D–F**). With progression of carcinogenesis from day 90 to 120, 4NQO intensified dysplastic changes with more atypical cells in multiple layers, resembling exophytic hyperkeratosis, arranged like honeycomb (**Figures 3C1, C2**). No severe dysplastic changes were noted in mice tongue of 4NQO+NLGP cohort (**Figures 3J–L**). The disease progresses along with days in 4NQO group, with thickened, condensed epithelium having keratin pearls (**Figures 3M1, M2**), however, extent of disease is significantly less in 4NQO+NLGP treated mice tongues. On day 180 onwards, a layer of cells was observed as being detached from the basement membrane, slowly migrating towards the muscular stroma and forming an island (**Figures 3G, M**). Similar observations have been observed on days 220 and 270 (**Figures 3H, I**). Interestingly, none of the changes have been seen in any mice of the 4NQO+NLGP cohort, though occasionally we observed a slight atypical hyperplasia of the tongue at late points (days 180 and 220). Tumors were visually observed after day 250 in 4NQO group (10/30) those were histopathologically confirmed as oral squamous cell carcinoma (**Figures 3N, O**). By tissue architecture, those carcinomas appeared as well differentiated squamous cell carcinoma, arranged in islands with varying shapes exhibiting the presence of numerous keratin pearls with larger sized acidophilic cells and pyknotic nuclei (**Figures 3N2**). Mice from 4NQO+NLGP (**Figures 3P–R**) cohort of same treatment period showed no visually or histopathologically detectable tumors. All changes are reported against a normal tongue (**Figure 3S**) at 4x magnification (S1) and 20x magnification (S2).

### 3.4 NLGP prevents progression of pre-neoplastic lesion by altering immune contexture during 4NQO mediated tongue carcinogenesis

**Figure 4:**
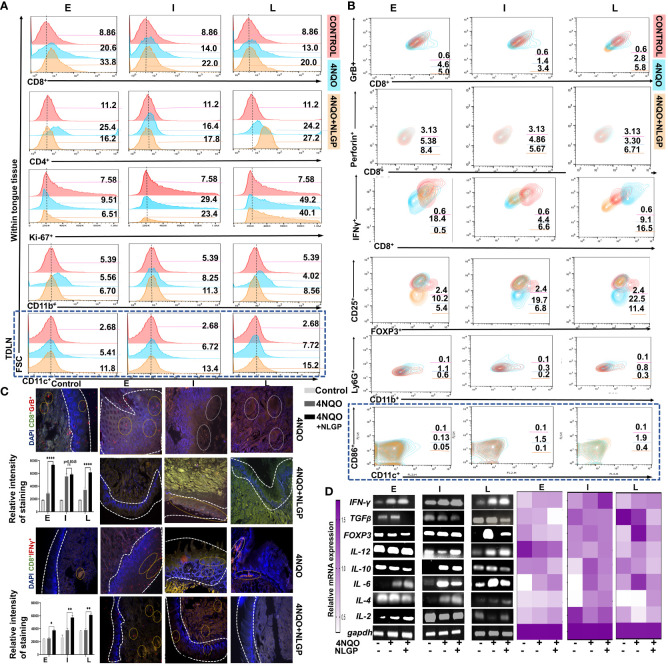
NLGP prevents progression of 4NQO induced pre-neoplastic lesions in tongues by modulating immune contexture. **(A)** Representative flow-cytometric histograms of immune cells (CD8^+^, CD4^+^, Ki-67^+^, CD11b^+^ and CD11c^+^) from mice tongues of 4NQO and 4NQO+NLGP treated cohorts at early, intermediate and late phases (n=9, in each group). **(B)** Contour plots representing CD8^+^IFNγ^+^, CD8^+^GrB^+^, CD8^+^Perforin^+^, CD4^+^CD25^+^FoxP3^+^, CD11b^+^Ly6G^+^ cells from tongues and CD11c^+^CD86^+^ cells within lymph nodes (blue highlighted area) from mice of 4NQO and 4NQO+NLGP treated cohorts in different phases (n=9, in each group). **(C)** Representative fluorescence images indicating presence of CD8^+^GrB^+^ and CD8^+^IFNγ^+^ T cells on tongues from normal, 4NQO and 4NQO+NLGP treated mice in three different phases. Dotted white lines separate the epithelium from tongue stroma. Positive staining and intensity analysis was performed on an average of 5 individual fields per section using Image J software. Bar diagrams obtained by two-way ANOVA following Tukey’s comparison test represent different relative gene expression levels in both 4NQO and 4NQO+NLGP cohorts. *p<0.05, **p<0.01, ****p<0.0001, ns, not significant. Normal distribution of the samples was ensured by performing normality and Log-normality tests. All of the data processed passed Shapiro-Wilks test for normal distribution. **(D)** Representative RT-PCR plots of pro- and anti-inflammatory cytokines from tongue single cell suspension at each phase of carcinogenesis. Heatmap depicting relative expression of individual genes compared to GAPDH.

The destruction of tumor cells is mostly dependent on CD8^+^ tumor infiltrating T lymphocytes (37). NLGP restricts murine tumor growth in CD8^+^ T cell dependent manner (1–3, 5). Here, we observed sequential reduction of CD8^+^ T cells from early to late phases of 4NQO carcinogenesis (**Figure 4A**); however, therapeutic NLGP replenishes this effector population in all phases. These CD8^+^ T cells also IFNγ^+,^GrB^+^ and perforin^+^ (**Figure 4B**). In contrast to CD8^+^ T cells, CD4^+^ T cells showed no significant change after NLGP treatment (**Figure 4A**). But importantly, immune-suppressor, CD4^+^CD25^+^Foxp3^+^ Treg cells, which showed a gradual increase with progression of dysplasia to carcinoma *in situ* in 4NQO cohort, is significantly reduced after NLGP treatment. Increased CD8^+^ T cells with decreased Tregs were correlated well with restricted tongue alterations in mice of 4NQO+NLGP cohort.

Given previous reports for strong immunomodulatory effect of NLGP in tumor transplanted model, next we studied the immune contexture of tongue tissues having 4NQO or 4NOQ+NLGP treatment. Since augmented CD8^+^T cell functions require antigen presentation, it remained essential to study the antigen presenting population. Thus, involvement of CD11b^+^ immune cells (preferably macrophage and/or neutrophil) and dendritic cells (DCs) (CD11c^+^) were explored. We observed CD11b^+^ cells were increased in tongues for 4NQO+NLGP cohort than 4NQO only at early time points (82%) followed by 73% and 47% in intermediate and late phases respectively (**Figure 4A**). However, CD11b^+^Ly6C^+^ myeloid-derived suppressor cells were decreased significantly in NLGP cohort than 4NQO only at all time points studied (**Figure 4B**). Interestingly, CD11c^+^ DCs showed an increased presence in TDLN from 4NQO+NLGP cohort than 4NQO group (**Figure 4A**; region marked in blue long dashed box); while a reverse condition was observed in tongue lesions *(data not shown)*. On analysis of CD11c^+^CD86^+^ DC population within mice lymph nodes, we observed a sequential increase in DCs from early to intermediate phases followed by a slowdown in late phase after 4NQO application. This population was increased further in the NLGP cohort that was observed most prominently in intermediate phases. Phasewise changes in individual immune cell population and immune portfolio are listed in **Supplementary Figures 2F–H**. To assess the proliferating ability of tongue derived cells, cells were stained with Ki67 antibody and in all phases number of proliferating cells was decreased in NLGP treated cohort (**Figure 4A**).

To understand whether the results obtained from mouse model bear any resemblance with human data, we compared results with the human database TIMER 2.0 (Tumor Immune Estimation Resource), which uses 10897 patient samples from 32 cancer types from ‘The Cancer Genome Atlas’ (TCGA database) to understand the effects of immune cells and their effects on HNSCC. This software categorizes HNSCC into 3 different groups: HPV independent (n=522), HPV negative (n=422) and HPV positive (n=98). Concordant to our results, we observed positive correlation between CD8^+^T cells and macrophages, in human HNSCC patients (**Supplementary Figures 3B, C**), which justifies the results obtained in this study from mouse model. Next, we seek to find out the cellular localization and expression levels of Granzyme B and IFNγ in the tongue tissues. Our fluorescent microscopic images indicate that both IFNγ and Granzyme B were increased and co-localized within CD8^+^ T cells in tongues from 4NQO+NLGP cohort (**Figure 4C**).

As chemical-insult induced inflammatory response that plays an important role in shaping up the altered tissue environment and critical in regulating trafficking and sustaining of immune cells, next we studied the expression of various cytokines at different time periods. RT-PCR analysis of affected tissues suggest that expression of IL-12 and IFNγ decreases post-day 60 with 4NQO administration but NLGP application significantly elevates their levels (**Figure 4D**) particularly observed at late time point after day 200 with application of 4NQO. Results suggest that IFNγ and TGFβ act reciprocally, while NLGP treatment augments IFNγ levels across each stage, TGFβ follows a reduction in comparison to the 4NQO counterparts. IL-2 appears to be reduced with 4NQO administration at early and late phases with moderate elevation in the intermediate phases, however, with NLGP treatment, expression of all these cytokines starts elevating and remained high enough till late phase. IL-6, IL-10 and TGF expression, on the other hand, significantly elevated from early time points and maintains higher expression with 4NQO treatment, and appears to be directly correlated with the carcinogenic progression and reduces after therapeutic intervention. With the help of our cellular level analysis and the fact that Tregs and monocytes influence IL-10 production, we might be able to link a decrease in IL-10 level to a downregulated trend nbsp;of Tregs after NLGP treatment. Interestingly, another immunosuppressive cytokine, IL-4 showed no significant change with 4NOQ treatment and a modest downregulation was observed after NLGP therapy.

### 3.5 NLGP prevents angiogenesis and epithelial to mesenchymal transition during 4NQO mediated tongue carcinogenesis

**Figure 5:**
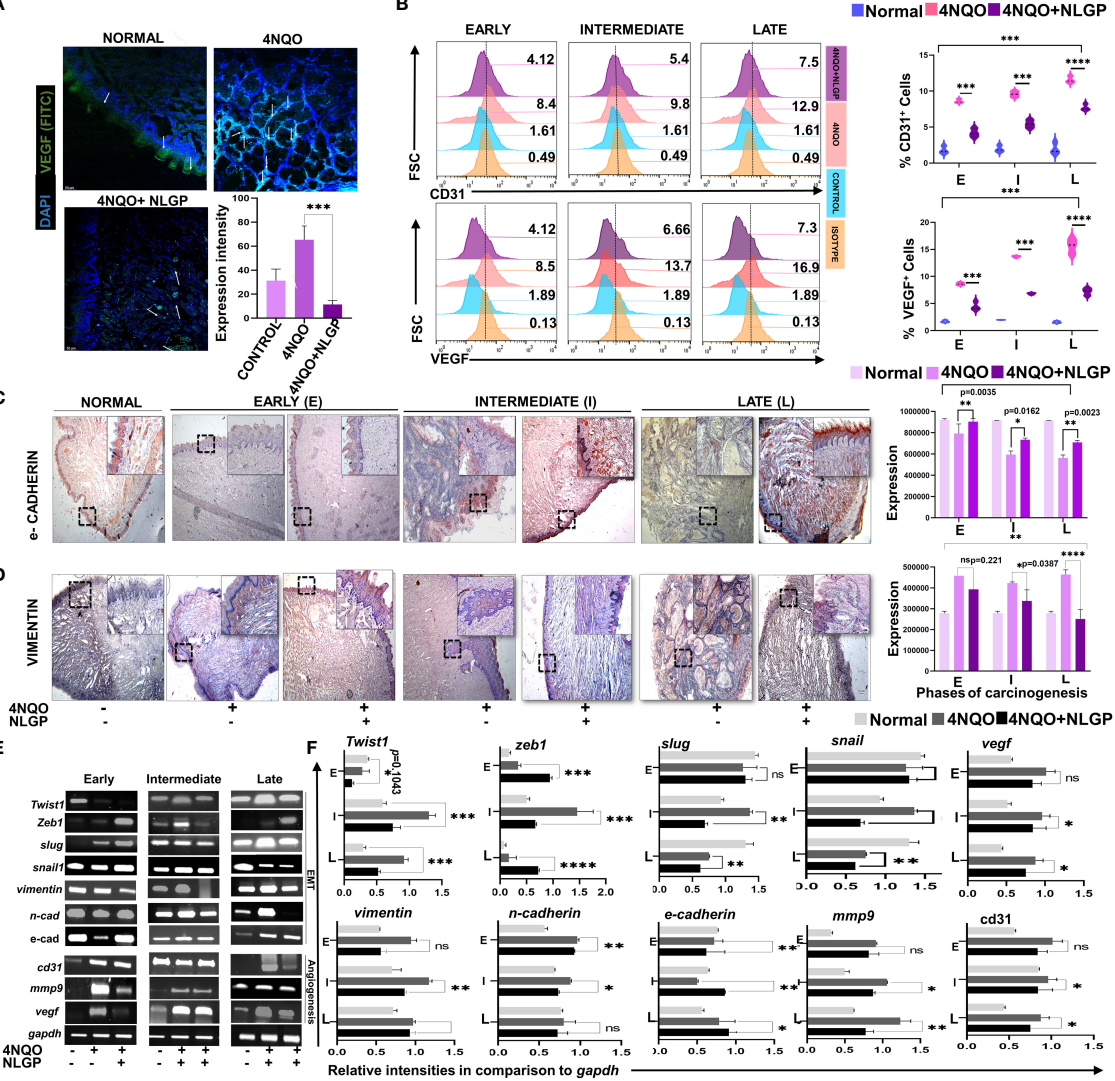
NLGP prevents angiogenic switch and EMT during 4NQO mediated tongue carcinogenesis. **(A)** Representative confocal images on VEGF expression in normal, 4NQO and 4NQO+NLGP treated tongues at late phase (Day 300). White arrows indicate areas of VEGF expression. Magnification 20x. Bar graphs obtained by 2 ways Anova test followed by Tukey’s multiple comparison test. Expression is measured as mean area of selected. **(B)** Flow-cytometric analysis of CD31 and VEGF within single cell suspensions of tongues from 4NQO and 4NQO+NLGP treated groups. Bars representing the expression level of CD31 and VEGF at various phases of carcinogenesis. **(C, D)** Immunohistochemical analysis of e-cadherin and vimentin on tongue epithelium at early, intermediate and late phases. Images were captured at 4x magnification; *inset* 20x. Bar graphs are depicting expression of staining intensity as an average of 5 individual fields. Analysis was done on Image J software. Significance values were obtained by 2 way Anova followed by Tukey’s multiple comparison test. **(E)** RT-PCR of EMT and angiogenesis related genes along with transcription factors, Snail, Slug, Zeb and Twist in mouse tongues. Data representative of experiments repeated in triplicate (n=3, at each time point). **(F)** Individual bar diagrams are obtained by two-way ANOVA following Tukey’s comparison test represent different relative expression levels of EMT and angiogenic genes in comparison to GAPDH in both 4NQO and 4NQO+NLGP groups. *p<0.05, **p<0.01, ***p<0.001, ****p< 0.0001, ns, not significant.

Given the importance of angiogenic switch during the progression and establishment of carcinogenesis (38), next we assessed the localization of VEGF expression within tongue tissue in the late phase of carcinogenesis (day 300) along with expression of VEGF and CD31, within tongue single cell suspension. Confocal microscopy of tongues at day 300 (end point) revealed intense VEGF expression in 4NQO cohorts, along the invasive zones but fairly lower expression in normal and 4NQO+NLGP cohorts (**Figure 5A**). Application of 24 times 4NQO over a 60 days period appeared sufficient to increase VEGF within tongues (**Figure 5B**), which also correlated with higher CD31 expression, a marker for neoangiogenic blood vessel formation, on a cellular level. On the other hand, NLGP showed significant restriction in tissue VEGF along with low CD31 expression especially at intermediate and late points (**Figure 5C**). Since EMT is the key hallmark of carcinogenesis progression, it remains extremely important to study localization of key EMT markers, e-cadherin and vimentin. Assessment of e-cadherin demonstrated a gradual loss of it from early to late phases of 4NQO treatment (**Figure 5C**), which was found to be restored after NLGP therapy. Conversely, the opposite trend was observed with vimentin expression and results demonstrated a prominent reduction in mesenchymal vimentin after NLGP treatment (**Figure 5D**), maximally at late phase (**Figure 5B**). 4NQO treatment showed upregulation of vimentin and n-cadherin after day 100, as detected at transcriptional level in concordance to the immune-staining results which was most prominent at later time-points with a modest downregulation of e-cadherin from early phase onwards (**Figure 5E**). Co-ordinately a higher expression of MMP7 (**Supplementary Figure 5A**) and MMP9 was observed with 4NOQO, especially at late phases (**Figure 5E**). Interestingly, NLGP mediated restricted carcinogenesis resulted significantly altered expression of EMT markers, and accordingly a lower expression of vimentin, MMP7, MMP9 was observed with a maintained expression of e-cadherin. To check the involvement of transcriptional regulators in the process of EMT, we analyzed master regulators, Snail, Slug, Zeb and Twist. Although the level of Zeb remains inconsistent, the level of Twist changes with different stages of carcinogenesis along with Snail and Slug. Levels of Twist remain minimal in the early phases of carcinogenesis but with progression, the Twist transcription is increased with carcinogen application, however, showed visible reduction with NLGP therapy. Twist along with Slug and Snail, interestingly showed an initial upregulation with NLGP treatment at the early phase of carcinogenesis, but started downregulating from intermediate points with NLGP treatment (**Figures 5E, F**; **Supplementary Figure 5B**). Since they are repressors of e-cadherin and are associated with tumor progression and invasiveness, the NLGP mediated normalization of e-cadherin and vimentin can easily be related to these findings and strongly confirms the ability of this drug as a potential EMT repressor.

### 3.6 NLGP interacts with Notch1 and Stat3 to restrict epithelial mesenchymal transition

**Figure 6:**
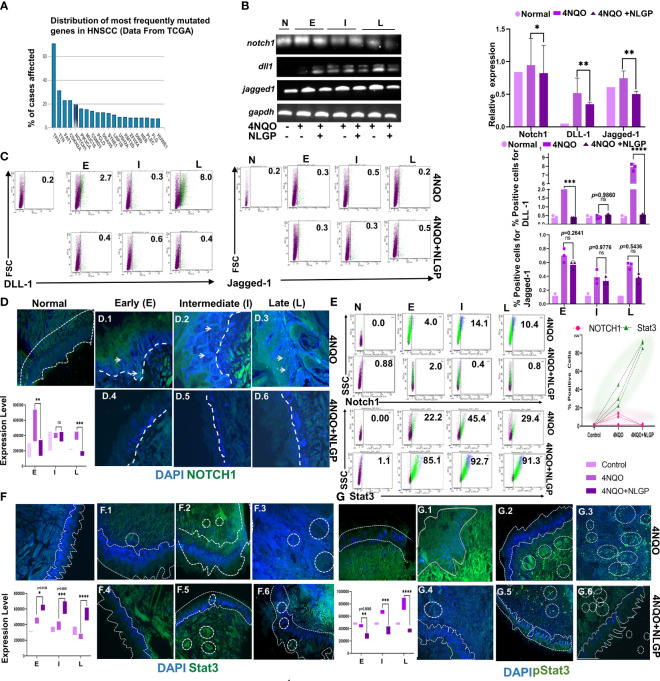
Altered NOTCH1/Stat3 signaling **(A)** A graph from TCGA representing the most commonly mutated genes in HNSCC. **(B)** Representative RT-PCR images of Notch1, DLL1 and Jagged 1 from normal, 4NQO and 4NQO+NLGP cohorts at early, intermediate and late points (Left) and bar graphs representing the relative intensity as compared to GAPDH (right). **(C)** Representative dot plots of DLL1 and Jagged-1 positive cells within single cell suspension of mice tongues of 4NQO and 4NQO+NLGP cohorts at three phases (n=3). Bar graphs plotted for DLL1 (top) and Jagged 1 (bottom) in Graphpad Prism. Significance was obtained by 2 way Anova followed by Tukey’s multiple comparison test. *p<0.05, **p<0.01, ***p<0.001, ****p<0.0001, ns, not significant. Fluorescence staining of Notch1 **(D)** with FITC (Green) in tongue sections from normal, 4NQO and 4NQO+NLGP treated mice at early, intermediate and late phases of carcinogenesis. Nuclei stained with DAPI (Blue). **(E)** Flow-cytometric staining of Notch1 (top panel) and Stat3 (bottom panel) in cell suspensions of carcinogen and therapeutically treated tongue (Left) and graph representing their expression levels (Right). n=3 for each phase. Fluorescence staining of Stat3 **(F)** and pStat3 **(G)** with FITC (green) and DAPI (nuclear) of tongues from normal, 4NQO and 4NQO+NLGP treated mice. Dotted lines indicate the margin of the epithelium. Circled areas indicate zonalised expression of various markers. Analysis of stained areas was performed on Image J software as a mean of 3 individual fields. Data are representative of two separate experiments, consisting of 6 mice per group in each case. Error bars indicate geometric mean ± SD. Analysis performed by 2-way ANOVA followed by Tukey’s Multiple comparison test on Graphpad Prism Software version 8.0.

Given the effect of NLGP on the EMT regulators and based on our earlier reports on the effect of NLGP on important regulators like STAT3 (11, 39), we now seek to investigate the potential role of NLGP on Stat3 along with Notch1. The candidature of Notch1 as an element of further study arose from a TCGA (Total Cancer Genome Atlas) database search on the most mutated genes for OSCC, which revealed Notch1 as one of the five most important signaling molecule for OSCC (**Figure 6A**). Alteration in the Notch1 gene is also associated with a panel of alterations in other genes (**Supplementary Figures 5A, C**) in OSCC patients. Higher levels of altered Notch1 also result in poor survival among OSCC patients (**Supplementary Figures 5B, E**). Based on the search results, we performed a transcriptional analysis to check the levels of Notch1 and two of its ligands, DLL1 and Jagged1 within the tongues of 4NQO and 4NQO+NLGP treated mice at different phases of carcinogenesis. Although the levels of DLL1 remain quite undefined in the earlier phases, prominent results were observed at late points, demonstrating a reduction with NLGP treatment (**Figure 6B**). This is in agreement with flow-cytometric studies on tongue single cells, where the maximum change was observed in the levels of DLL1 at the late point (From 8·0 to 0·6) of NLGP treatment (**Figure 6C**). Although the changes of Jagged 1 levels remain insignificant, results clearly indicate possible involvement of DLL1 towards the Notch1 alterations. Notch 1 reportedly is correlated positively with matrix metal proteases in a variety of cancer types. We hence performed some microscopy based studies to find out the levels and localization of these molecules. Our studies indicate Notch1 expression being more diffused on the membrane and in the cytoplasm than in the nucleus in these OSCC samples. Strong membranous Notch1 expression was often observed at the border of cancer nests at late point. Moreover, a significant decrease in Notch1 expression was detected in NLGP treated tongues as compared to 4NQO group (**Figure 6D**). Furthermore, studies demonstrate considerable lowering of Notch1 expression within tongue single cells after NLGP therapy (**Figure 6E**, top panel). The maximal amount of shifts in Notch1 was found to be in the intermediate phases of carcinogenesis between days 100 to 200.

Given the fact that STAT3 phosphorylation remains a prognostic marker for EMT in various carcinomas (40), we needed to determine the location and expression status of this molecule in the tongues during the carcinogenic process. Stat3 activation is one of the early key events in initiation of OSCC. STAT3 has been positively linked to mainly Grade 3 and Grade 4a OSCC human patients as per data obtained from GEPIA 2.0 (Gene Expression Profiling database (**Supplementary Figure 5A**). Higher level of STAT3 in OSCC patients is also correlated negatively with their overall survival (**Supplementary Figure 5B.4**). Although STAT3 levels were found to be increased than normal from early to late points (**Figures 6E** (lower panel), **F**) and subsequently getting elevated in the NLGP cohort from early to middle phases (**Figure 6F**) with almost similar levels at late points. An intriguing finding was that, in the NLGP cohort, high pSTAT3 expression, particularly at intermediate and late points after 4NQO treatment, was significantly reduced to almost minimal level at all phases after NLGP treatment (**Figure 6G**). Thus, in conjunction with our transcriptional results involving Snail, Slug and Twist, a lower pSTAT3 level with NLGP can be linked to the level of the EMT transcriptional factors.

### 3.7 NLGP modulates crosstalk between different proteins involved in 4NQO mediated tongue carcinogenesis

**Figure 7:**
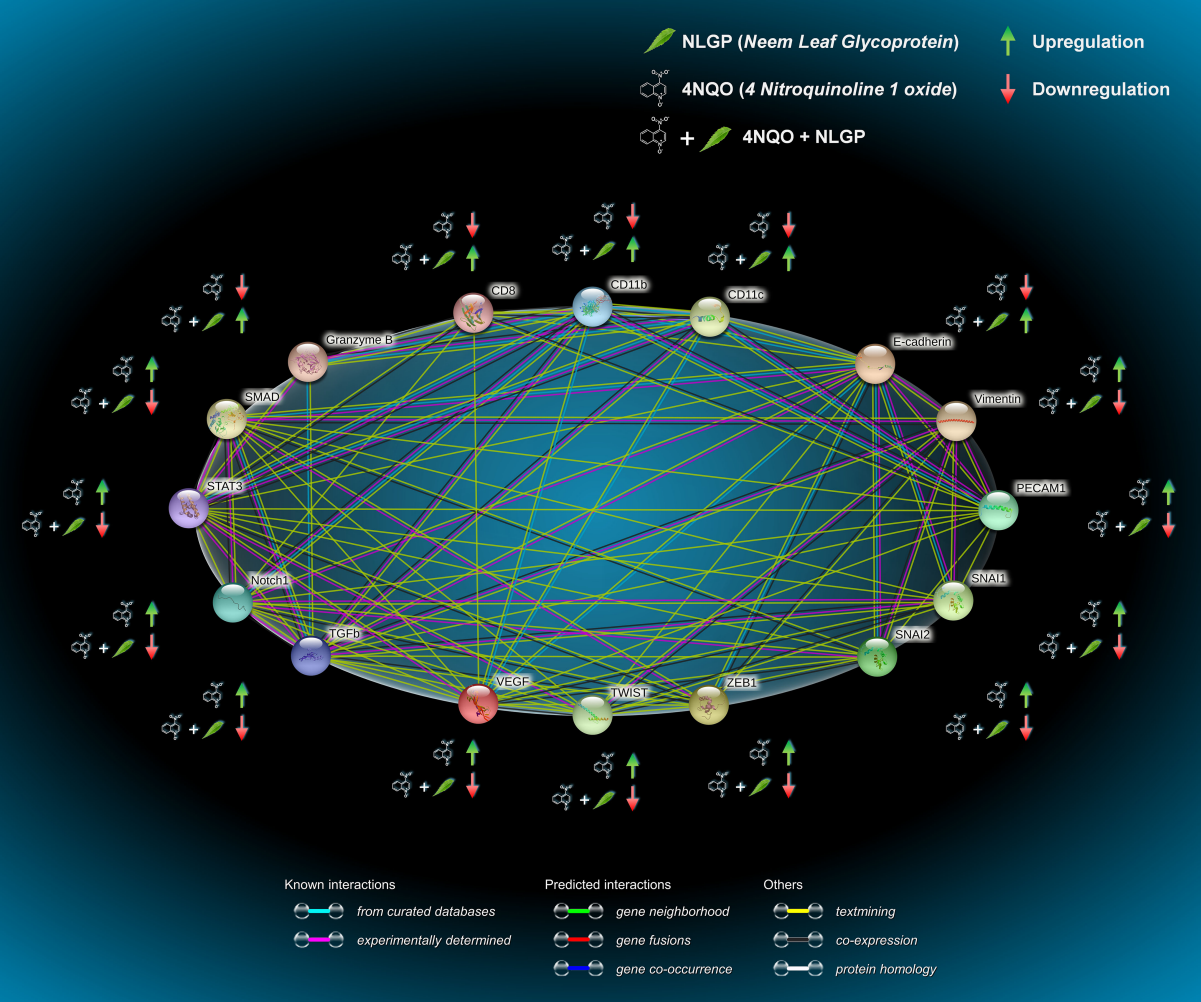
Functional protein association network analysis module shows functional association of different proteins of study and their alterations along with 4NQO and 4NQO+NLGP treatment via String Module (Version 11.5). All nodes are colored indicating all proteins are known and structure already identified. Lines indicate the different degree of associations between them as obtained by various searches each represented by a different color. Sky blue lines, known interaction from curated data bases; pink lines, experimentally determined; green lines, predicted interaction of gene neighborhood; red lines, gene fusion; blue lines, gene co-occurrence. Colored nodes, query proteins and first shell of interactors; Green arrow indicate an overall upregulation and downregulation is indicated by red arrow. The different degree of associations between each protein is given in **Supplementary Table 3**.

To understand the interactions between all the different proteins involved in this study, we used database STRING, which relies on the annotated proteomics maintained by Swissprot. Functional protein association network analysis module shows functional association of different proteins of study and their alterations along with 4NQO and 4NQO+NLGP treatment via String Module (Version 11.5). All molecules involved in this study are mutually related to each other with varying degrees of correlation, (**Figure 7**; **Supplementary Table 3**) under normal conditions. With application of 4NQO and 4NQO+NLGP, their expression has been altered (Upregulated expression marked in green arrows, and downregulated expression in red arrow). Taken together, this Figure provided a relationship structure and their alterations in normal, 4NQO and 4NQO+NLGP cohorts between various immune cells in relation to EMT and their effects on various signaling parameters.

## 4 Discussion

A decade later of the establishment of immunomodulatory properties of NLGP, therapeutic application of this glycoprotein in murine sarcoma solid tumor model was reported in 2013. Such *in vivo* study not only demonstrated the tumor growth restriction by NLGP, but also documented the significant increase in survival of tumor hosts. In agreement with the accumulated evidences on NLGP’s immunomodulation, here, curtain has been raised to show the NLGP mediated CD8^+^ T cell dependent immunomodulation, which is the key reason of sarcoma growth restriction and increased survivability (3). The report from transplanted sarcoma tumor was subsequently confirmed on melanoma (1), carcinoma (2) and lymphoma models, where tumor heterogeneity was maintained without demonstration of sequential initiation-promotion protocol, as observed in human carcinogenesis process. Thus, the demand is urgent to appraise NLGP’s efficacy during slow carcinogenic process with features of hyperplasia, dysplasia, *in situ* carcinoma and invasive carcinoma.

To fulfill such critical objective thorough study on animal model having resemblance with human cancer is prerequisite. Oral cancer model was chosen for this particular study. Oral carcinogenesis can be achieved by the local delivery of the carcinogen, most often by labor-intensive methods including brushing the hard palate, tongue or gingiva with concentrated water solution of the carcinogen. Among several established oral carcinogens and mouse models, DMBA had been widely used in hamster cheek pouches to mimic the sequential steps of human oral carcinogenesis. However, this model is not widely used as the cheek pouch has a different etiology and genetic constituent as that of humans (41). Another model is 4NQO induced tongue carcinogenesis that closely mimics the sequential steps of tobacco induced human oral carcinogenesis (42). Areca-nut/betel-leaf/tobacco chewing habits are widely prevalent in many parts of Asia, including various parts of India. Many betel-quid products are used in different forms by different communities and ultimately these products come in close contact of oral mucosa. Relationship of betel-quid/tobacco habit and cancer of various parts of the oral cavity is widely discussed (17). Hence, this model is used in this study to assess the mechanism of action of NLGP in sequential process of oral carcinogenesis, more specifically tongue carcinogenesis that have never been evaluated.

In agreement with earlier reports, we also observed sequential events of carcinogenic process, e.g., hyperplasia, dysplasia, carcinoma *in situ* etc, in 4NQO initiated oral carcinogenesis, thus, appeared as right model to study the intervention of NLGP through the process of carcinogenesis. Optimization of the immune functions is now considered as an appropriate modality of cancer remedy. In this path, the search for a novel rehabilitative and natural method of immunomodulation leads us application of the glycoprotein from the leaves of *Azadirachta indica* (Neem), which has been proved to exhibit plausible antitumor effects in mice models of sarcoma, melanoma, carcinoma and lymphoma and reported throughout the last decade (1–12). NLGP may fulfill the dearth necessity of a treatment modality, which causes an upliftment of the host immune system without associated complications, as observed in conventional treatment modes.

This study was conducted with a total of 270 Swiss albino female mice distributed in three groups of 90 each. The overall study was spaced over three experimental sets, where we observed squamous cell carcinoma in 84.44% (76/90) mice (**Table 1**) (in the 4NQO group alone across early, intermediate and late phases of carcinogenesis). This rate however goes down significantly (almost 30%), when NLGP was administered s.c. once a week for 4 weeks, followed by monthly injection of NLGP (25μg). Of the 90 mice in the NLGP cohort, we observed restoration/normalcy in the features in 70% (63/90) (**Table 2**) mice. The positive effect of NLGP was also prominent in the later phases of carcinogenesis post day 200, similar to the middle and late phases. NLGP treated mice exhibited higher body weight and increased survivability as compared to their normal counterpart. They also display normal facial features, absence of lesions or damaged tongue architecture. Histological images of mouse tongue treated with 4NQO revealed several characteristic features as basal cell hyperplasia, loss of papillae, formation of rosette structures and carcinoma cell nest with progression of oral carcinogenesis. We report for the first time that NLGP majorly preserves the normal architecture of the mouse tongues in most cases even after repeated administrations of 4NQO. While neoplastic changes along with papillae loss appeared in the early phase of 4NQO induced carcinogenesis, it is interesting to observe that throughout the early phase, the NLGP treated tongues maintain the normal architecture even after 4NQO painting. With the exception of 27 out of 90 mice, all mice of NLGP cohort displayed normal tongue features macroscopically as well as microscopically.

In order to delineate the mechanism of oral carcinogenesis preventive functions of NLGP, first we examined the overall immune landscape of 4NQO painted tongues, keeping unique immunomodulatory properties of NLGP in mind. Initially effort has been given to elucidate the alteration in profiles of T cells, regulatory T cells, dendritic cells, macrophages, NK cells and others in 4NQO painted mice tongues having *in vivo* exposure of NLGP. NLGP does not have any oncolytic effect; it is an interesting immunotherapeutic molecule efficient to drive type 1 immune bias. All the effects of this molecule have been studied earlier in transplanted tumor models, however the role of NLGP in the initiation progression model of carcinogenesis have never been established. Analysis of single cell preparation of tongues revealed a substantial increase in the cytotoxic CD8^+^ T cell population in NLGP treated cohorts since early phases of carcinogenesis, with no significant alterations in CD4^+^ T cells. Enhanced cytotoxicity of this particular type of T cells is confirmed by the detection of presence of IFNγ, granzyme B and perforin on these cells, as detected in both cellular and transcriptomal levels. NLGP mediated release of IFNγ from cytotoxic T cells during their killing activity was reported earlier along with upregulated expression of granzyme B and perforin on T cells (3, 5). Ultimate creation of type 1 immune environment by NLGP helps in cancer immunosurveillance that may take part an important role in delaying 4NQO induced tongue tumorigenesis. NLGP also slowed down the proliferation of malignant tongue cells as detected by lower expression of Ki67 proliferative marker.

As we have seen activation of cytotoxic T cells in 4NQO painted tongues after NLGP treatment and it is expected that these activated cells are participating in delayed carcinogenesis process by NLGP. To unravel the process of T cell activation, we checked CD11b^+^ and CD11c^+^ macrophages and dendritic cells respectively, chiefly involved in antigen presentation process. A steady increase in CD11b^+^ macrophage population was detected from early through late phase, suggesting their active participation in CD8^+^ T cell activation process. Here, nature of CD11b^+^ cells unexplored, but NLGP mediated steady conversion from M2 to M1 macrophages was documented in our earlier study (39). We assume positive impact of NLGP driven CD11b^+^ cells in 4NQO mediated carcinogenesis process. In this context, it is worthy to mention that role of NLGP driven M1 macrophages in CSC niche alteration during 4NQO mediated tongue carcinogenesis is currently observed (*unpublished observation*; Bera et al.).

In an attempt to check the status of professional antigen presenting dendritic cells, we look out for CD11c expression within the mouse tongue single cell preparation and observed no significant difference following NLGP treatment. However, an interesting observation was the identification of CD11c^+^ cells within tumor draining lymph nodes exhibiting increasing trend from early to late phases of 4NQO mediated carcinogenesis with NLGP treatment. Flow-cytometric data on CD11c^+^CD86^+^ cells confirmed the migration of dendritic cell population from the damaged tongue to the adjoining lymph node.

As we acquired evidence of cytotoxic functions of CD8^+^ T cells with required antigen presentation from macrophages and dendritic cells, next, we attempted to explore the status of suppressor cells that may regulate the CD8^+^ T cell functions. Our study showed significant increase in the suppressor Treg population from early to late phases after 4NQO treatment. Extent of increase was high in early phases than others favoring 4NQO mediated tongue carcinogenesis. However, NLGP therapy drastically decreases the proportion of CD4^+^Foxp3^+^ cells irrespective of phases. Here, we observed prominent role of suppressor Treg cells in 4NQO carcinogenesis that was remarkably downregulated by NLGP. Similarly, MDSCs were also participated in this process, but only modest changes were noted with NLGP. Both of these NLGP’s action helps to withdraw the suppression on T cells, thereby, altered tongue cells may be eliminated to retard the carcinogenic process.

4NQO mediated tongue carcinogenesis also deregulate pro-inflammatory and anti-inflammatory cytokine milieu as expected to favor the carcinogenic process. NLGP actively modulate most of the altered functions, like, upregulation of IFNγ, IL-2, prominently in intermediate and late stages. NLGP mediated downregulation of TGFβ and others are also noted to create tumor microenvironment that hinders tumor growth.

Both angiogenesis and EMT play crucial role in the tumor progression, thus, their correlation with oncogenesis is of great value. Our studies on angiogenesis and EMT have revealed reduction in angiogenic markers (CD31 and VEGF) in the transcriptional and cellular levels in carcinogen exposed mice with NLGP therapy. The anti angiogenic effects of NLGP are most profound in the early and middle phases upto day 200. Immunohistochemical analysis of key EMT markers (e-cadherin and Vimentin) show phasewise alteration in 4NQO cohort with normalization in NLGP group. Collectively membranous loss of e-cadherin, with an aberrant expression of vimentin is an important key feature of malignancy (43). The hallmark of EMT process is loss of cell-cell adhesion and increased cell motility by the downregulation of e-cadherin and upregulation of mesenchymal type vimentin. This process known as “cadherin switching” appears to provoke cancer cell migration and invasion (44) and has always been associated with more aggressive and less differentiated malignant cells.

Wang et al. (PMID: 29018057) (45) have reported promising inhibition in 4NQO -induced oral squamous cell carcinogenesis with anti-PD1 antibody. Delaying in carcinogenesis is also observed here by us with NLGP. Anti-PD1 treatment is widely discussed immune check point inhibitor (ICI) therapy and its efficacy in various human cancer has been proved and presently in clinic (46). However, its efficacy is limited. Reason of limited success of anti-PD1 therapy is discussed recently by us (47). This therapy works by inhibiting PD1-PDL1 interaction to potentiate T cell activity. NLGP also works by activating CD8^+^ T cells, but its role in inhibition of PD1-PDL1 interaction is not evaluated. But, NLGP can delay the generation of exhausted T cells. Contrary to the high cost of anti-PD1 antibody, NLGP is expected to be low cost, thus, NLGP therapy will be economical and may add newer view over existing anti-PD1 therapy, particularly in countries with poor socio-economy.

## 5 Conclusion

Collectively, our study has provided strong evidence that NLGP has got an important role in restriction of oral carcinogenesis with initiation of NLGP therapy at early phase of carcinogen exposure. Since this is a time dependent study of 300 days, involving constant exposure to the carcinogen, it is expected that the immune equilibrium is disturbed throughout. Robust immunomodulatory power of NLGP maintains the normal immune functions particularly by CD8^+^ T cell based elimination of carcinogen altered cells. After completion of four therapeutic doses of NLGP, single booster dose in every month is required to maintain the cytotoxic T cell pool throughout. Still, our earlier findings regarding increased mice survivability due to NLGP therapy would have great translational significance. Considering its safe usage and ease of administration, combined with its notable effects on optimization of immune microenvironment, angiogenesis and epithelial-mesenchymal transition, future studies can be aimed for further development of the molecule towards clinics.

## Data Availability

The original contributions presented in the study are included in the article/**Supplementary Material**. Further inquiries can be directed to the corresponding authors.

## Supplementary material

Supplementary Table 1 List of materials and antibodies for this study.

Supplementary Table 2 List of PCR primers.
