# Corrigendum: The immunomodulatory impact of naturally derived neem leaf glycoprotein on the initiation progression model of 4NQO induced murine oral carcinogenesis: a preclinical study

**DOI:** 10.3389/fimmu.2024.1470330

**Published:** 2024-11-18

**Authors:** Juhina Das, Saurav Bera, Nilanjan Ganguly, Ipsita Guha, Tithi Ghosh Halder, Avishek Bhuniya, Partha Nandi, Mohona Chakravarti, Sukanya Dhar, Anirban Sarkar, Tapasi Das, Saptak Banerjee, Sandip Ghose, Anamika Bose, Rathindranath Baral

**Affiliations:** ^1^ Department of Immunoregulation and Immunodiagnostics, Chittaranjan National Cancer Institute, Kolkata, India; ^2^ Department of Oral Pathology, Dr. R. Ahmed Dental College and Hospital, Kolkata, India; ^3^ Department of Pharmaceutical Technology (Biotechnology), National Institute of Pharmaceutical Education and Research (NIPER), Sahibzada Ajit Singh Nagar, Punjab, India

**Keywords:** 4NQO, CD8^+^ T cells, immunotherapeutics, neem leaf glycoprotein, Notch1, oral carcinogenesis, Stat3, epithelial mesenchymal transition

In the published article, there was an error in the legend for **Figures 1** and **2** as they have been interchanged.

The corrected legend appears below.

**Figure 1:**
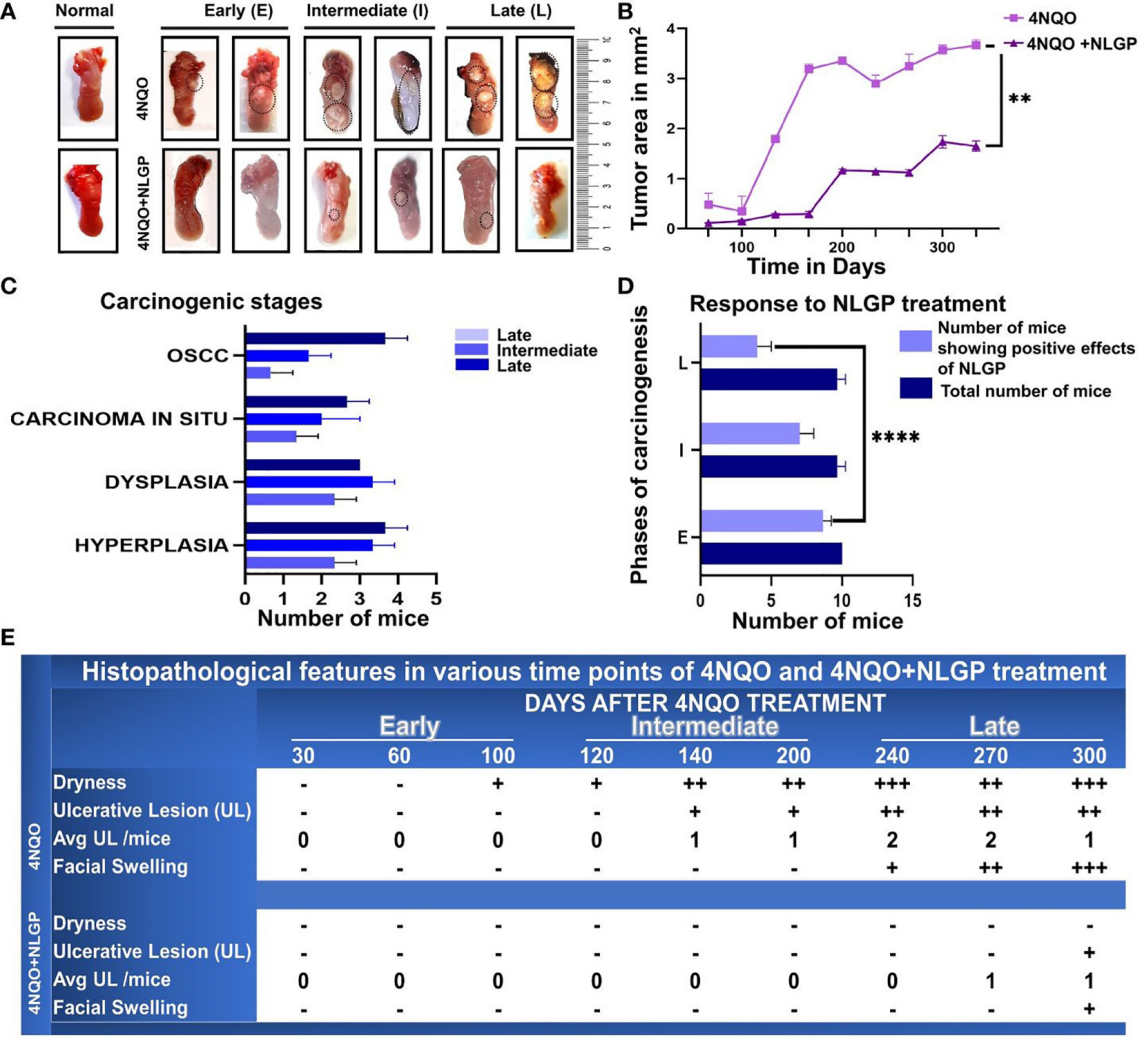
NLGP maintains macroscopical tongue features during 4NQO mediated carcinogenesis. **(A)** Representative images of mouse tongues at early (day 1- day 100), intermediate (day101-day 200) and late stages (day 201- day 330) of 4NQO and 4NQO+NLGP treated tongues. Dotted areas indicate the presence of lesions. **(B)** Line graphs representing the mean lesion area in mm2. Data presented as mean ± SD. **(C)** Bar diagram representing total number of mice collectively from the experimental (4NQO) group, displaying different stages of carcinogenesis in each of early, intermediate and late phases (n=30), inclusive of all phases, experiment repeated thrice.**(D)** Total number of mice that was benefited by NLGP treatment in early, intermediate and late phases n=3 at each time point. **(E)** Table indicating changes appearing in mouse tongues and faces at different phases of carcinogenesis. **p< 0.0001,+ minimally present, ++ present in moderate levels, +++ present in high levels, -, absent.

**Figure 2:**
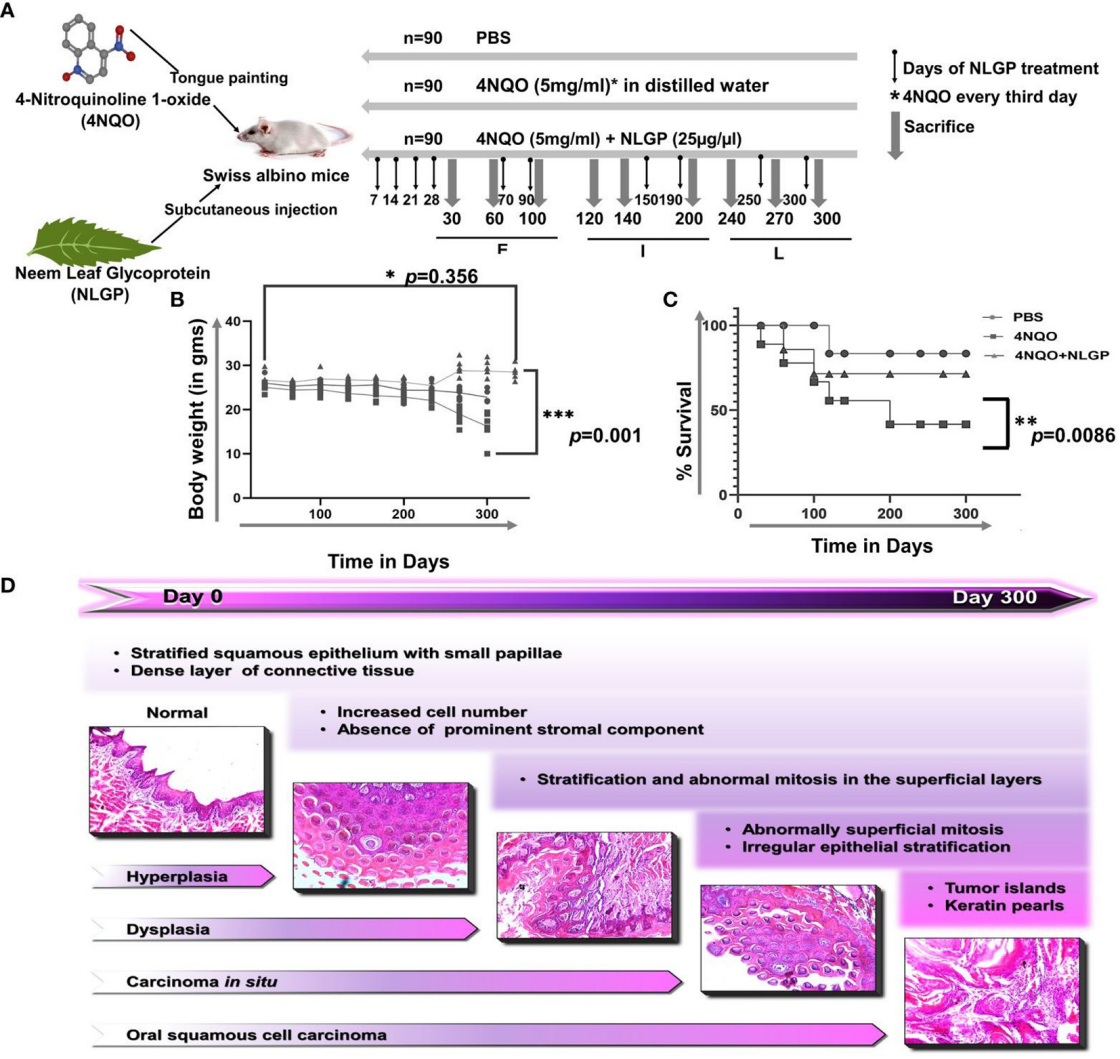
NLGP protects detoriation of physical features and body weight loss during 4NQO mediated tongue carcinogenesis. **(A)** 4NQO, NLGP and treatment protocol. Experimental design is showing 4NQO and NLGP treatment. 4NQO was applied by tongue painting every third day and NLGP was given subcutaneously (25µg/mice) on day 7, 14, 21 and 28. Thereafter, NLGP dose was repeated on days 100, 150, 200, 250 and 300. **(B)** Graphical representation of body weight changes after 4NQO painting with and without NLGP treatment, n=9 per group. Analysis was done by 2 Way ANOVA followed by Tukey’s multiple test. **(C)** Kaplan Meyer’s survival analysis for control, 4NQO and 4NQO+NLGP treated mice. Significance was obtained by Log-rank (Mantel-Cox) test. **p=0.0086. **(D)** Representation of sequential carcinogenesis stages from day 0 to day 300. H&E images were captured on 20x magnification. *p<0.001.

A correction has also been made to **Abstract**, ***Methods***. This sentence previously stated:

“After five consecutive treatments with 4NQO (starting Day7), one group of mice was treated with NLGP (s.c. 25µg/mice/week)”

The corrected sentence appears below:

“After three consecutive treatments with 4NQO (starting Day7), one group of mice was treated with NLGP (s.c. 25µg/mice/week)”

The authors apologize for these errors and state that they do not change the scientific conclusions of the article in any way. The original article has been updated.

