# Supplementary material for: The immunomodulatory impact of naturally derived neem leaf glycoprotein on the initiation progression model of 4NQO induced murine oral carcinogenesis: a preclinical study
Source: Front Immunol. 2024 Mar 22;15:1325161. doi: 10.3389/fimmu.2024.1325161 (PMC10996442; doi:10.3389/fimmu.2024.1325161)
Supplement: Supplementary Table 1 — List of materials and antibodies for this study. [file DataSheet1.pdf]

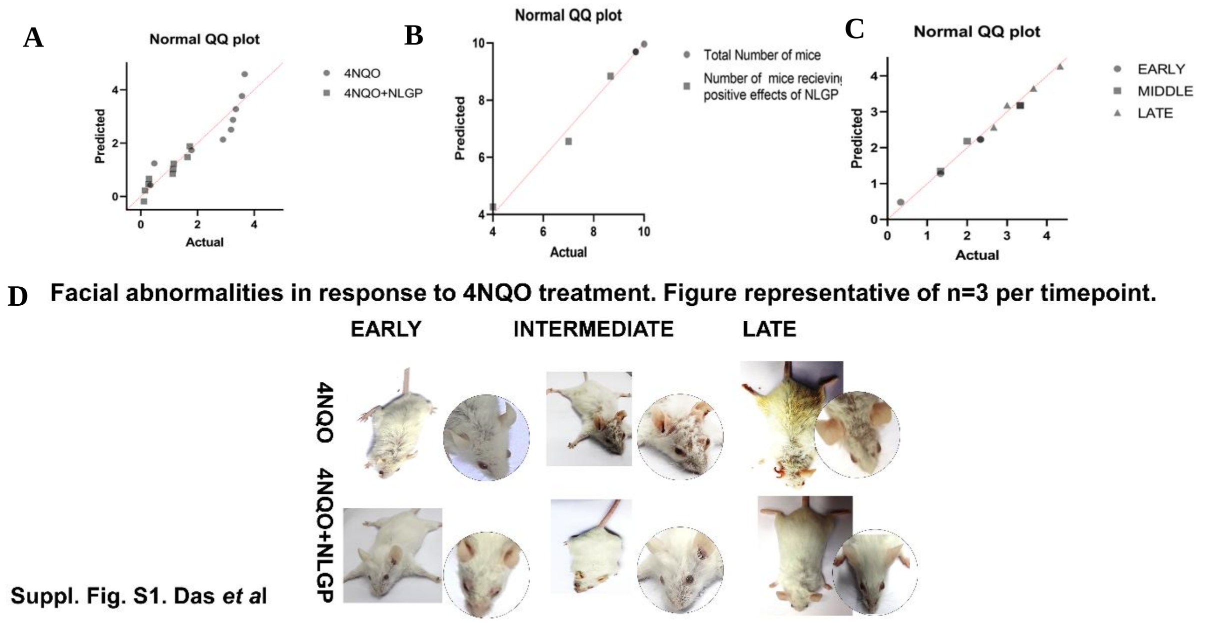

Supplementary Fig.1: (**A, B, C)**: Q-Q plots indicative of normally distributed data of 1B,1C and 1D respectively. (**D).** Representative images of facial abnormalities in response to 4NQO and 4NQO+NLGP treatment.

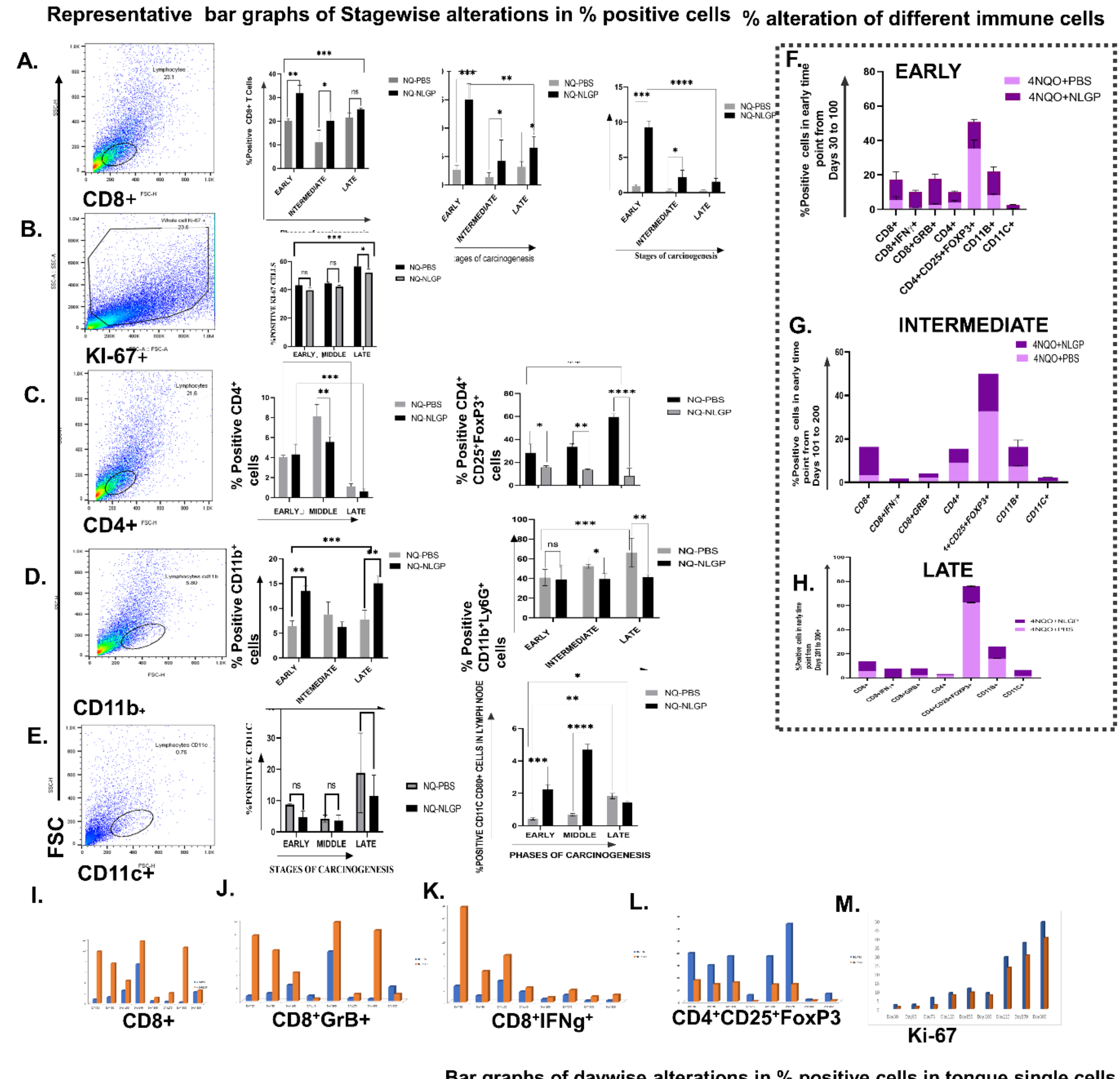

Suppl. Fig. S2. Das *et al*

Supplementary Fig. 2: (A-E). Gating strategies for different immune cells on 4NQO, 4NQO+NLGP treated mouse tongues. Graphs represent changes for each immune cell type at each of the different carcinogenic phases. **(F-H).** Combined changes for each immune phenotype at all the three phases respectively.(**I**). Flow-cytometric data of perforin expressing cells gated on CD8^{+}T cells at each of early, intermediate and late phases with and without NLGP treatment (n=9 per phase, repeated thrice). Right, Comparative bar graph representation for the same. (**J-O**).Day wise change for various cells with and without NLGP treatment. Graphs were plotted on Graphpad Prism software version 8.0. Bar diagrams obtained by two-way ANOVA following Tukey's comparison test. n=9 per phase, *p<0.05, **p<0.01, ****p<0.0001, ns: not significant.

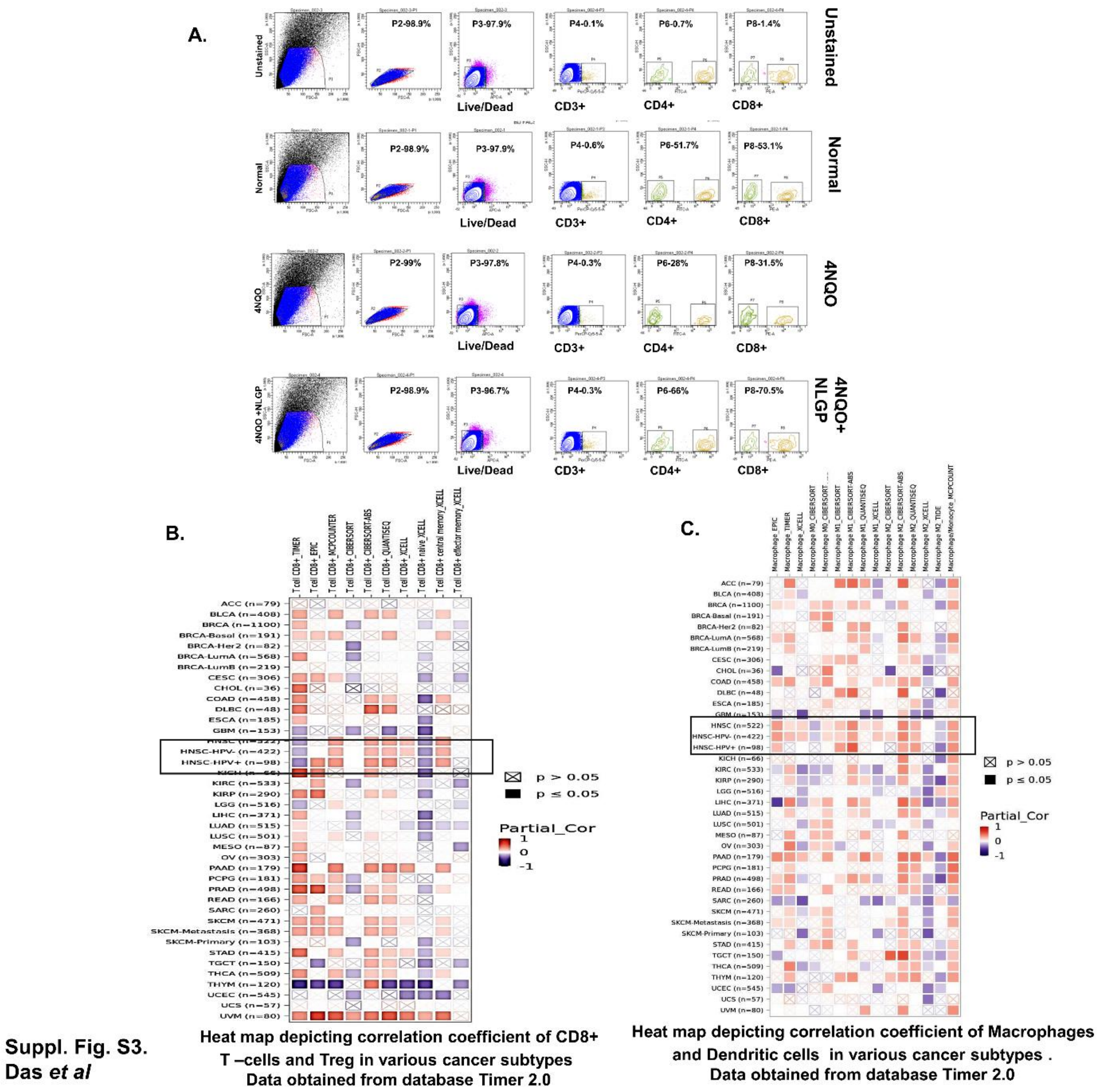

Supplementary Fig. 3: (**A**). Representative flow cytometric plots of $CD3^+$ $CD4^+CD8^+$ lymphocytes within tongue single cells at late points. P2- gated lymphocyte zone, P3- Live cell population within the gated zone (blue), P4- Total population of CD3+ Positive cells within the gated region. P6,P8- Percentage of CD4+ and CD8+ population respectively within P3. Data representative of 3 separate experiments. Data acquired and analysed on BD LSRFortessa ™ cell analyser **(B).** Heatmap representing correlation of $CD8^+$ T cells in different human cancers. Highlighted area indicates HNSCC. HNSCC (n=522), HPV^+ HNSCC, (n=98) and HPV^- HNSCC (n=422) human patients. (**C**). Heatmap representing correlation of macrophages in human HNSCC (n=522), HPV^+ HNSCC, (n=98) and HPV^- HNSCC (n=422) human patients. Data source – Timer 2.0.

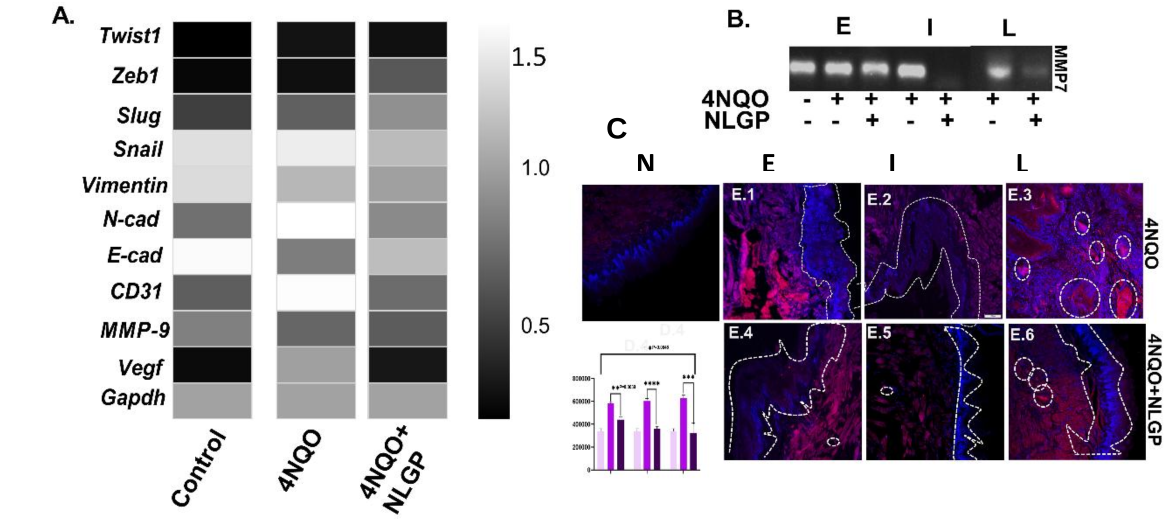

Suppl. Fig. S4. Das *et al*

Supplementary. Fig S4: (A) Heatmap representative of the relative intensities of various angiogenic transcription factors and EMT genes of Fig 5 in comparison to GAPDH (**B)**: RT-PCR of MMP7 from mouse tongue. (**C)** Immunofluoresent imaging of MMP9 within tongue single cells. Representative images of n=3 in each of early (E), intermediate (I) and Late (L) time points.

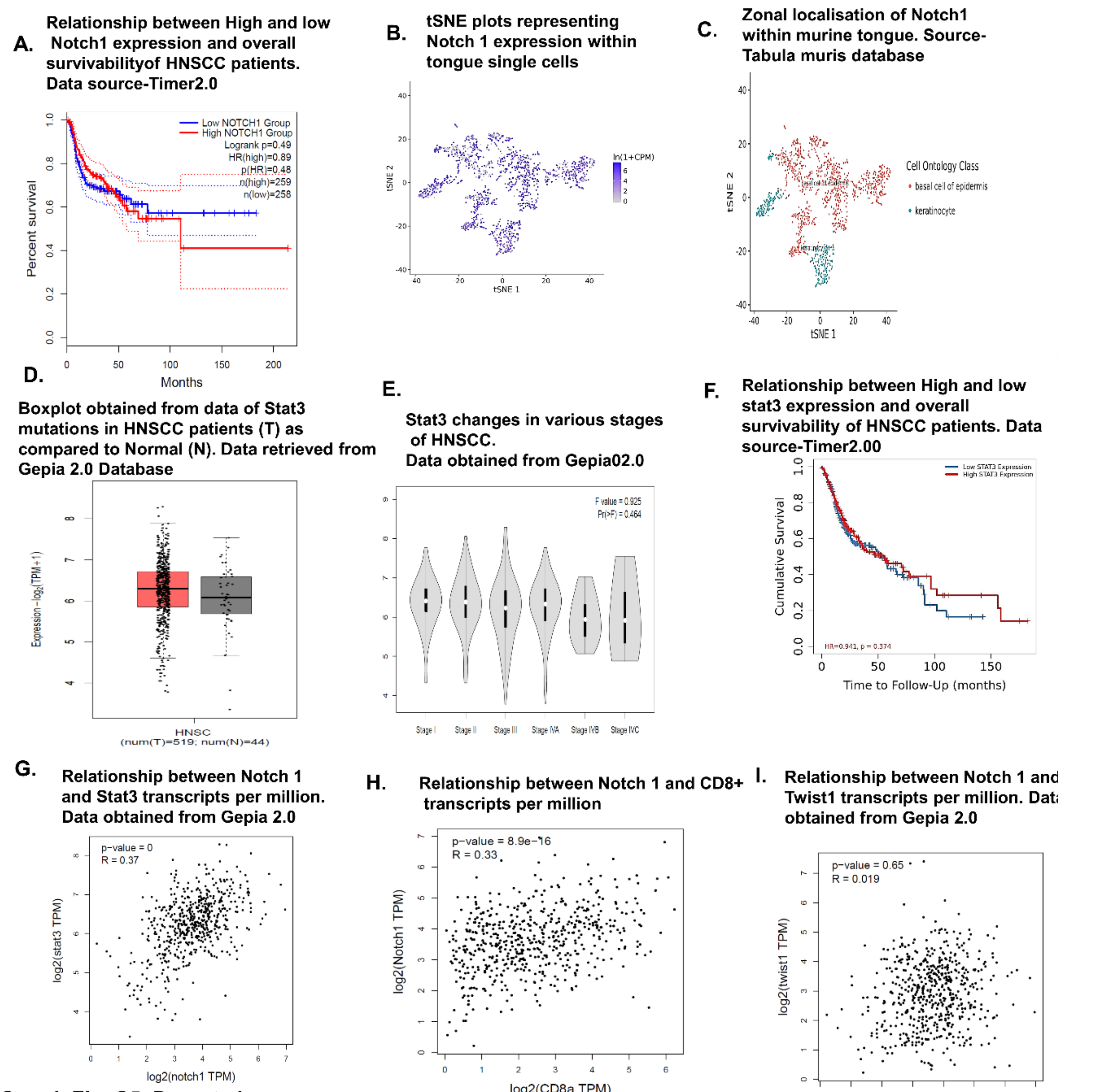

Suppl. Fig. S5. Das *et al*

Supplementary Fig.5: (A). Survivability curve (in months) in relation to high and low Notch 1 mutations in human HNSCC patients. **(B).** tSNE plots of Notch 1 expression in murine tongue sample analysed flow-cytometrically and (**C**)**.** tSNE plot indicating distribution of Notch 1 within basal cells of epidermis and keratinocytes of mouse tongue. Data obtained from Tabula muris database. (**D**)**.**Box plots obtained from data on Stat3 mutations in Normal (n=44) and HNSCC (n=519) patients. (**E**)**.**Violin plots representing Stat3 changes in various stages of HNSCC**. F.**Survivability curve representing high and low Stat3 expression in human HNSCC patients. Data Source:Timer 2.0 (**G-I**): Log fold changes of Notch 1 with respect to Stat3, Twist1 andCD8 in human HNSCC patients. Data Source: Gepia 2.0

Table 1: Number of mice affected by 4NQO treatment in experimental (4NQO) and **t**herapeutic (4NQO+NLGP) groups

	**Early**		**Intermediate**		**Late**	
**Carcinogenic features**	**4NQO** 30 (n=10 per repeat)	**4NQO + NLGP** 30 (n=10 per repeat)	**4NQO** 30 (n=10 per repeat)	**4NQO + NLGP** 30 (n=10 per repeat)	**4NQO** 30 (n=10 per repeat)	**4NQO + NLGP** 30 (n=10 per repeat)
**Hyperplasia**	9	1	5	1	2	2
**Dysplasia**	11	3	6	3	9	1
**Carcinoma *in situ***	5	0	8	3	5	4
**OSCC**	1	0	5	0	10	2
**Total affected mice at the end of all experimental repeat in each phase**	**26**	**3**	**24**	**7**	**26**	**9**

Table 2. Number of responding mice to NLGP therapy

**Phases of Carcinogenesis**	**Total number of mice**			**Mice responded to NLGP therapy**		
Early	10	10	10	10	8	9
Intermediate	10	10	10	8	8	6
Late	10	9	10	8	7	5
**Total**	**30**	**29**	**30**	**26**	**23**	**20**
